# Mitochondrial dysfunction and oxidative stress in OSAHS and associated comorbidities: Mechanism and potential targeted therapeutic strategies

**DOI:** 10.1016/j.redox.2026.104409

**Published:** 2026-09-23

**Authors:** Linyi Zhong, Yiwen Guo, Jiaxuan Zong, Xiushan Yang, Jiaxing Sun, Yinwen Zhang, Jiyuan Fan, Yuanhe Feng, Siwen Wang, Yining Wang, Ke Du, Mengmeng Wang

**Affiliations:** a Department of Otolaryngology Head and Neck Surgery, Shengjing Hospital, China Medical University, Shenyang, Liaoning, 110122, China; b Department of Ultrasound, Shengjing Hospital of China Medical University, Shenyang, Liaoning, 110004, China; c School of Pharmacy, Department of Pharmacology, China Medical University, Shenyang, 110122, China

**Keywords:** Oxidative stress, Obstructive sleep apnea-hypopnea syndrome, Mitochondrial dysfunction, Oxidative damage, Multisystem comorbidities

## Abstract

Obstructive sleep apnea-hypopnea syndrome (OSAHS) is associated with multisystem comorbidities, including cardiovascular disease, metabolic disorders, and neurological, pulmonary, and kidney diseases. Current studies have revealed that the pathophysiology of OSAHS and its associated comorbidities is complex. However, chronic intermittent hypoxia-induced mitochondrial dysfunction and oxidative stress are considered to play an important role. In recent years, mechanisms of interaction between mitochondrial dysfunction and oxidative stress have gained attention and have emerged as important potential therapeutic targets. Future delivery systems may improve the tissue or mitochondrial targeting of investigational agents, although their efficacy and safety in OSAHS remain to be established. This review highlights the mutual reinforcement and positive feedback loop between mitochondrial dysfunction and oxidative stress, and provides an overview of the current research on associated therapeutic drugs, their novel delivery systems, and development potential. Our review aims to provide new ideas and methods for the treatment of OSAHS-associated comorbidities.

## Highlights

• CIH triggers mitochondrial dysfunction and oxidative stress in OSAHS.
• These two processes mutually reinforce each other, forming a positive feedback loop.
• This vicious cycle drives the progression of OSAHS-associated multi-system comorbidities.
• This review summarizes preclinical drugs targeting mitochondrial dysfunction and oxidative stress in OSAHS.

## Graphical abstract

**Figure:**
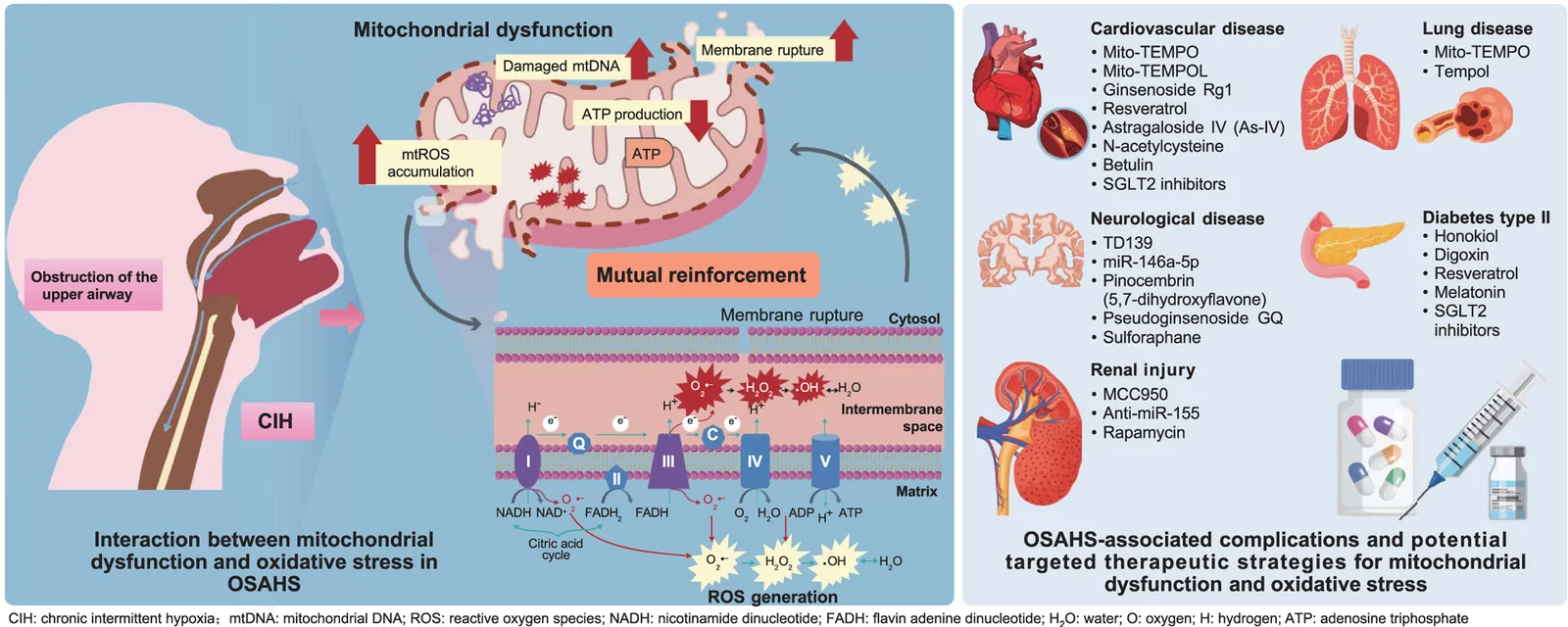

## 1 Introduction

Obstructive sleep apnea-hypopnea syndrome (OSAHS) is a common sleep disorder affecting 1 billion individuals aged 30–69 years worldwide and is observed predominantly in males [1]. OSAHS is characterized by repetitive collapse of the upper airway during sleep, resulting in chronic intermittent hypoxia (CIH) and hypercapnia. This results in sleep fragmentation, thereby increasing respiratory effort, recurrent nocturnal arousals, and sympathetic nerve activity [2]. OSAHS-associated comorbidities include cardiovascular disease [3,4], metabolic disorders [3], and neurological [5], pulmonary [6], and kidney [7] diseases. Currently, clinical treatments for OSAHS and associated comorbidities involve behavioral therapy, continuous positive airway pressure (CPAP), and surgical treatment; however, limitations including surgical risks and recurrence probability remain [[8], [9], [10]]. Although no radical pharmacotherapy for OSAHS is available, some drugs may improve symptoms in OSAHS, such as aroxynbutynin and atomoxetine [11,12]. The efficacy of current drugs is limited, which may stem from their imprecise target of action. Therefore, further in-depth research is essential to clarify its precise target and thereby break through the therapeutic bottleneck.

Recent research suggests that mitochondrial dysfunction and oxidative stress play important roles in the pathophysiology of OSAHS, providing a potential theoretical basis and research direction for the development of novel interventional strategies targeting this disease. Mitochondrial dysfunction and oxidative stress are key factors in the development of OSAHS and related complications [13,32,41]. Repeated breathing apnea is the main characteristic of OSAHS. It is similar to repeated ischemia-reperfusion events and leads to increased reactive oxygen species (ROS) [42], which are natural products of the mitochondrial respiratory chain [43]. Hypoxia facilitates the production of mitochondrial ROS (mtROS) by inhibiting mitochondrial respiration [44]. Oxidative stress arises when ROS synthesis exceeds antioxidant defenses [41,45,46]. The mitochondria-targeted antioxidant alleviates CIH-induced dysfunction, including swelling, mtROS release, and mPTP opening [13]. The central pathological axis linking these comorbidities is CIH-induced mitochondrial injury, which triggers ROS overproduction and oxidative stress, ultimately driving multisystem damage.

Drugs targeting mitochondrial dysfunction and oxidative stress, such as antioxidant drug Mito-TEMPO, the traditional medicinal Chinese herb Astragaloside IV, and N-acetylcysteine (NAC), have demonstrated protective effects in experimental CIH models of OSAHS-associated comorbidities. Furthermore, vitamins B6, B12, C, E, folic acid, alpha-lipoic acid, and Coenzyme Q can potentially alleviate specific cognitive and cardiovascular damages linked to OSAHS [47]. Hence, interventions targeting mitochondrial impairment and oxidative stress may provide a rationale for future therapeutic development.

This narrative review was based on a literature search of PubMed and Web of Science using combinations of terms related to OSAHS/CIH, mitochondrial dysfunction, oxidative stress, and associated comorbidities. Additional searches were performed for specific therapeutic agents. The search covered publications from 2000 to 2026, with a few earlier foundational studies retained where no updates existed. Both preclinical and clinical studies were included, prioritizing direct CIH evidence. Moreover, we comprehensively list existing and previous treatments targeting mitochondrial impairment and oxidative damage to potentially alter OSAHS development.

## 2 Mitochondrial dysfunction and oxidative stress

### 2.1 Mitochondrial dysfunction

Most eukaryotic cells have mitochondria that serve as the primary center of cellular energy production. They play a crucial role in lipid and amino acid metabolism, iron synthesis, calcium (Ca^2+^) regulation, and several inflammatory signaling pathways [48]. Mitochondria are double-membrane organelles with the outer mitochondrial membrane (OMM) and the inner mitochondrial membrane (IMM), which separates the mitochondria into two spaces: the inter-membrane space (IMS) and the matrix. The OMM contains membrane proteins involved in mitochondrial transport and apoptosis. Cholesterol is predominantly localized to the OMM. It engages in lipid exchange with mitochondria via endoplasmic reticulum-mitochondria contact sites, contributing to the maintenance of mitochondrial membrane integrity and the regulation of lipid metabolism [49]. The IMM stores mass proteins involved in the oxidative phosphorylation (OXPHOS), glycolysis, and tricarboxylic acid (TCA) cycles [50]. Cardiolipin is almost exclusively localized to the IMM, where it plays an indispensable role in core mitochondrial functions, including maintaining bioenergetics, regulating dynamics, and mediating processes such as apoptosis and the response to oxidative stress [51,52].

The mitochondrial electron transport chain (ETC) utilizes multiple electron transfer reactions to generate high-energy phosphate carriers (ATP) through OXPHOS [53]. Proton movements from the mitochondrial matrix into the IMS, along with electron transfer, create the mitochondrial membrane potential (ΔΨ), which generates ATP and acts as a prime indicator of mitochondrial function [54]. Cardiolipin binds to the ETC protein complexes on the IMM via electrostatic interactions, thereby significantly improving electron transfer efficiency [51]. Mitochondrial dysfunction is characterized by bioenergetics impairment, loss of ΔΨ, ROS production imbalance, mitochondrial dynamics disorders, and cell death [55].

Cellular bioenergetics and metabolism are closely correlated with mitochondria. Adverse environmental conditions such as hypoxia, inflammation, and hyperglycemia dysregulate mitochondrial respiratory activities, cause excessive mtROS production, loss of ATP production, and accumulation of damaged mitochondrial DNA (mtDNA) [56]. The mitochondrial permeability transition pore (mPTP), a transmembrane protein complex in the IMM, is activated by excessive ROS production, triggering a sudden alteration in permeability. This causes ΔΨ collapse and mitochondrial membrane rupture, leading to mtROS production. The continuous ROS generation initiated by mtROS has been defined as “ROS-induced ROS release” (RIRR) [57]. The opening of mPTP facilitates particle outflow from the mitochondrial matrix and releases cytochrome *c* (Cyt *c*), Ca^2+^, and ROS, further decreasing ATP synthesis and regulating cell death [58].

Mitochondrial biogenesis, mitophagy, and mitochondrial dynamics maintain the required number and quality of mitochondria. Mitochondrial biogenesis drives mitochondrial proliferation and mtDNA replication during cellular stress and division in response to cellular requirements for energy, as well as mitochondrial quality control (MQC) mechanisms [59]. This process is upregulated by the peroxisome proliferator-activated receptor gamma coactivator 1-alpha (PGC-1α) [60]. Both physiological and environmental factors, such as hypothermia, starvation, inflammation, and oxidative stress, may induce mitochondrial biogenesis [61]. Mitophagy is a selective autophagic process that eliminates damaged or excess mitochondria, playing a vital role in regulating mitochondrial health, quantity, and cellular balance [62]. Mitophagy has two modes: receptor mitophagy and PINK1/Parkin-dependent mitophagy, where the latter is the primary pathway for eliminating damaged mitochondria [63]. Mitochondrial dynamics involves mitochondrial fission and fusion and maintains homeostasis of the mitochondrial network through a series of processes [50]. Mitochondrial fission generates many smaller mitochondria, performs a physiological role in mitochondrial redistribution and proliferation, and separates defective mitochondrial fragments from healthy mitochondria, further boosting clearance by mitophagy [64]. It is primarily induced by dynamin-related protein-1 (Drp1) oligomerization, which is activated by fission protein 1 (Fis1) and mitochondrial fission factor (Mff), resulting in accumulation of Drp1 from cytoplasm to OMM surface and fragmentation of OMM [65]. Various stimuli such as hypoxia, hyperglycemia, excess Ca2^+^, high adenosine 5′-monophosphate (AMP) levels, and/or high levels of ROS upregulate mitochondrial fission via Drp1 activation [64]. Mitochondrial fusion is a dynamic cellular mechanism by which smaller mitochondria merge to form larger, more robust structures. This process is crucial in maintaining mitochondrial balance by allowing defective mitochondria to integrate with healthy components, thereby revitalizing impaired organelles and restoring their membrane potential. Moreover, it acts as a quality control system, ensuring mitochondrial integrity and function are preserved [66]. Mitofusin 1 (Mfn1) and mitofusin 2 (Mfn2) are located on the OMM, tether to adjacent mitochondria, and regulate OMM fusion. The optic atrophy 1 (OPA1) protein located on the IMM induces its fusion and cristae remodeling [67]. Cardiolipin in the IMM induces membrane curvature via its distinctive structure, directly interacts with the inner membrane fusion protein OPA1 to regulate its activity, and consequently drives mitochondrial inner membrane fusion [68]. Mitochondrial fusion is upregulated by cytosolic signals involved in the initial phase of apoptosis, mitotic progression, or cellular senescence [64]. Mitochondrial dynamics is disrupted due to stress conditions, which increases mitochondrial fission but decreases mitochondrial fusion. This is accompanied by dysfunctional mitochondrial accumulation, which can worsen mitochondrial function, ultimately resulting in substantial cellular abnormalities and a variety of disorders [69].

Mitochondria and the endoplasmic reticulum are tightly interconnected organelles, with extensive physical contact sites existing between them, known as mitochondria-ER contact sites (MERCSs). MERCSs serve as a major site for the non-vesicular transport of lipids such as cholesterol and a critical hub coordinating mitochondrial dynamics remodeling and quality control. On the one hand, MERCSs provide precisely positioned nodes for mitochondrial fission and fusion, where the associated molecular machinery assembles and operates in an organized manner. On the other hand, under hypoxia, FUNDC1—a key regulator of mitophagy—is activated via dephosphorylation at MERCSs, where it recruits Drp1 and other factors to drive mitochondrial fission, subsequently mediating the autophagic clearance of damaged mitochondrial fragments. Thus, in response to hypoxia, MERCSs facilitate a programmed “fission-clearance” response, synergistically regulating mitochondrial morphology and quality, and thereby constituting a core adaptive mechanism for cellular survival under low-oxygen conditions [70].

Activation and regulation of cell death, including apoptosis and pyroptosis, can be induced by mitochondrial dysfunction. Mitochondria play a crucial role in the initiation of the apoptotic phase. Upon exposure to oxidative stress or other apoptotic stimuli, the OMM undergoes increased permeability, leading to the release of pro-apoptotic proteins, Cyt *c*, and mitochondrial DNA into the cytoplasm [71]. Cyt *c*, which is typically anchored to the inner mitochondrial membrane via cardiolipin, dissociates and is released into the cytosol due to oxidation-induced changes in its affinity for cardiolipin. The released Cyt *c* subsequently activates the apoptosome, initiating the caspase cascade and ultimately resulting in apoptosis [72,73]. Pyroptosis is a tightly controlled mechanism of programmed cell death that hinges on the inflammasome pathway. This triggers caspase production, which consequently activates the gasdermin proteins, namely GSDMD and GSDME. This activation leads to the formation of N-terminal GSDMD (NT-GSDMD), which travels to the cell surface and oligomerizes to create a pore resulting in lysis and the release of inflammatory cytokines [74]. The process of oligomerization relies on mtROS [75]. NT-GSDMD/E can target mitochondria and stimulate Cyt *c* release, resulting in enhanced mitochondrial apoptotic pathways [76]. Additionally, NT-GSDMD also damages both the inner and outer mitochondrial membranes, leading to mitochondrial damage and enhanced pyroptosis [77].

### 2.2 Oxidative stress

Oxidative stress is characterized by an imbalance between the production and neutralization of ROS. This impairs nearly all indispensable intracellular macromolecules and results in DNA, protein, and tissue damage [78]. Studies have demonstrated that oxidative stress is a commonality among various chronic diseases, including cancer, metabolic, cardiovascular, lung, and neurological diseases [79]. ROS include molecules from O_2_, including superoxide (•O_2_−), hydrogen peroxide (H_2_O_2_), hydroxyl radical (•OH), hydroxyl (OH^−^), peroxide (O_2_^2^−), and singlet oxygen. ROS can originate from endogenous and exogenous sources, where exogenous production occurs due to exposure to radiation, smoking, alcohol usage, and some medicines, whereas endogenous ROS production includes mitochondria, the ER, the NADPH oxidase (NOX) family, and peroxisomes [80]. Mitochondria are the major site of endogenous ROS production [81]. OXPHOS generates ATP as well as ROS. ER-associated stress likely activates redox signaling mediators during ROS formation in the ER [82]. Among these, ROS are primarily generated by the oxidoreductases Ero1α and PRDX4, which are localized in the ER lumen, during the process of protein disulfide bond formation [83]. Peroxisomes are cellular organelles responsible for metabolic reactions, such as fatty acid β-oxidation, and for detoxifying harmful peroxides. Within the process of fatty acid β-oxidation, the enzyme acyl-CoA oxidase 1 (ACOX1) catalyzes substrate oxidation, directly generating H_2_O_2_. These ROS are primarily confined to the peroxisomal matrix [83]. The NOX family located on the plasma membrane serves as a major source of intracellular ROS. These enzymes transfer electrons from cytosolic NADPH across the membrane to the extracellular space or endosomal lumen, thereby generating •O_2_− [84]. ROS at low to moderate levels function as chemical messengers, playing a key role in various cellular processes. Conversely, when ROS levels increase to moderate to high levels, they damage proteins, DNA, and protective cellular layers, a condition known as oxidative stress [85]. Therefore, ROS homeostasis is crucial for cellular function.

ROS homeostasis plays important roles in cell proliferation, differentiation, apoptosis, and necrosis [86]. Organisms have developed antioxidant systems that are primarily dependent on enzymatic and low-molecular-weight antioxidants to maintain ROS homeostasis [87]. The defense against oxidative stress relies on a network of antioxidants, including key enzymes such as superoxide dismutase (SOD), catalase (CAT), glutathione peroxidase (GPx), glutathione reductase (GR), thioredoxin (Trx) system, and peroxiredoxin (Prx), as well as smaller antioxidant molecules such as vitamins C and E, along with glutathione (GSH) [[88], [89], [90]]. In humans, SOD exists in three primary variants: zinc-dependent SOD1, manganese-dependent SOD2, and extracellular SOD3, all of which play a crucial role in converting superoxide radicals into hydrogen peroxide [91]. ROS clearance in the cytoplasm and mitochondria is primarily carried out by SOD, GPx, Prx, Trx, and GSH, whereas in peroxisomes, it relies mainly on CAT with support from the Prx and GSH systems [92,93]. GR catalyzes the reduction of oxidized glutathione (GSSG) to reduced GSH in both the cytosol and mitochondria, thereby maintaining the stability of the cellular reduced glutathione pool and ensuring the sustained and effective functioning of GPx and other GSH-dependent antioxidant and detoxification pathways [94]. Beyond directly scavenging reactive oxygen species (ROS), the antioxidant defense system also regulates cellular stress responses through redox signaling. Upon oxidative stress, the transcription factor nuclear factor erythroid 2-related factor 2 (Nrf2) is released from its inhibitor Keap1, translocates into the nucleus, binds to the antioxidant response element (ARE), and activates the expression of antioxidant enzymes, thereby enhancing cellular antioxidant capacity [94]. Typically, ROS levels are maintained by antioxidant defense systems to ensure physiological ROS signaling for cellular function. When ROS homeostasis is impaired, excessive ROS can cause cell damage resulting in various diseases (Fig. 1).

**Fig. 1:**
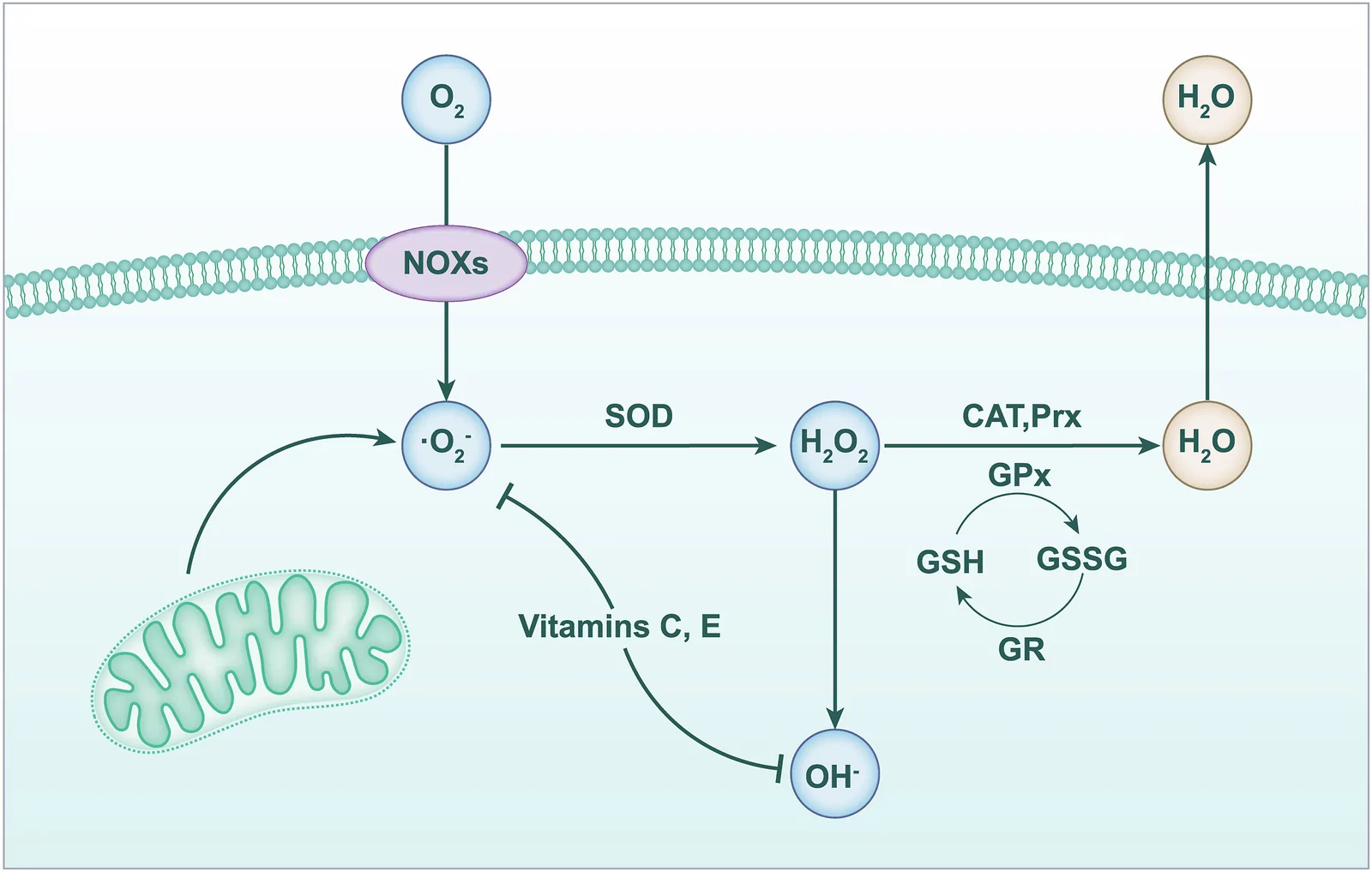
The balance between ROS generation and the antioxidant defense system.

### 2.3 Interactions between mitochondrial dysfunction and oxidative stress

Mitochondrial malfunction causes oxidative stress, which causes mitochondrial malfunction, including mitochondrial membrane potential depolarization, mitochondrial metabolism, and mitochondrial dynamics dysfunction (Fig. 2). Among them, abnormal accumulation of mitochondrial metabolites (such as succinate and NADH) can further exacerbate oxidative stress by interfering with electron transport chain function and altering cellular redox homeostasis. The interaction between the endoplasmic reticulum and mitochondria can lead to a further aggravation of oxidative stress (Fig. 3).

**Fig. 2:**
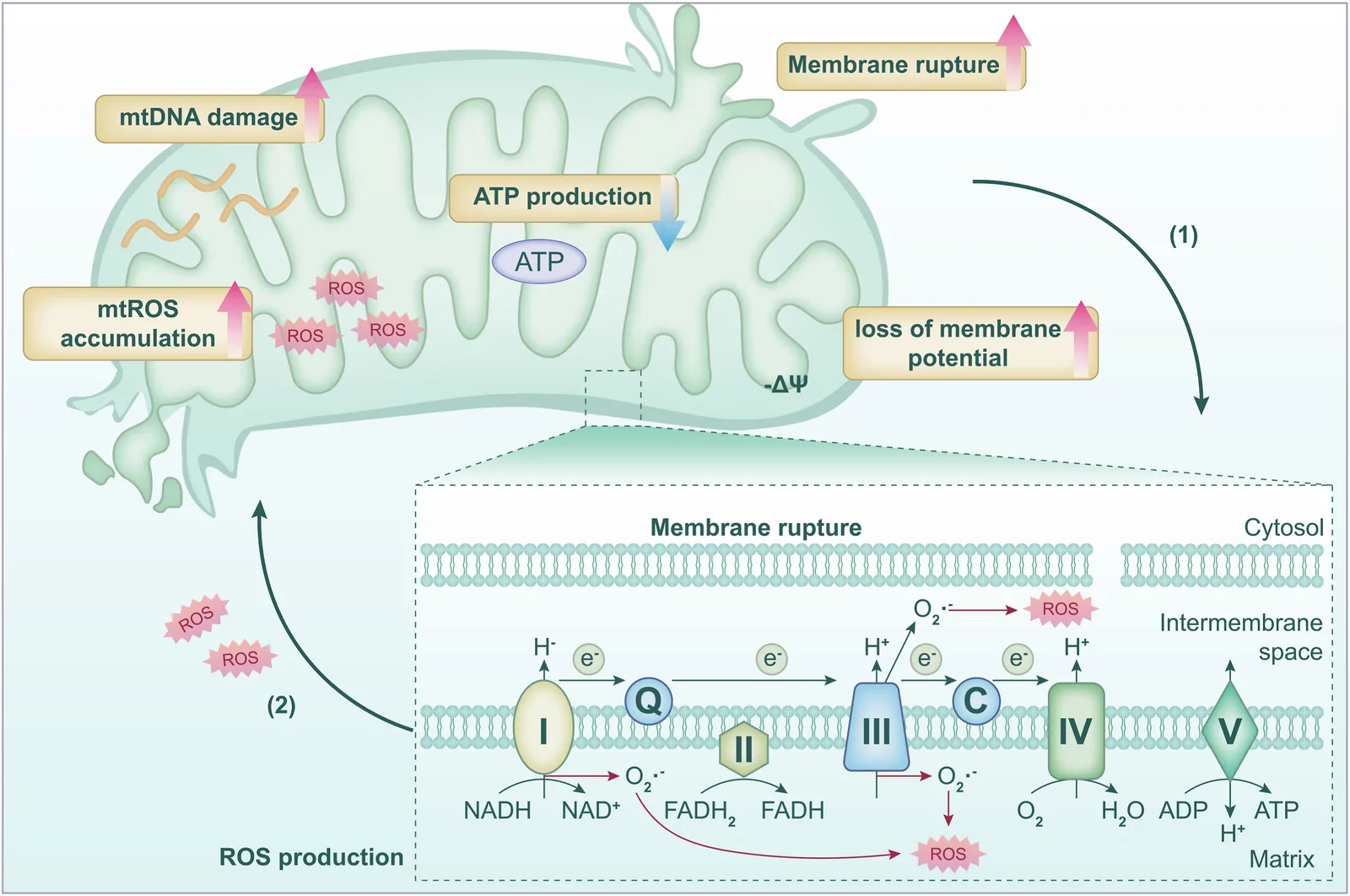
Interactions between mitochondrial dysfunction and oxidative stress. (1) Mitochondrial dysfunction causes oxidative stress. (2) Oxidative stress induces mitochondrial dysfunction.

**Fig. 3:**
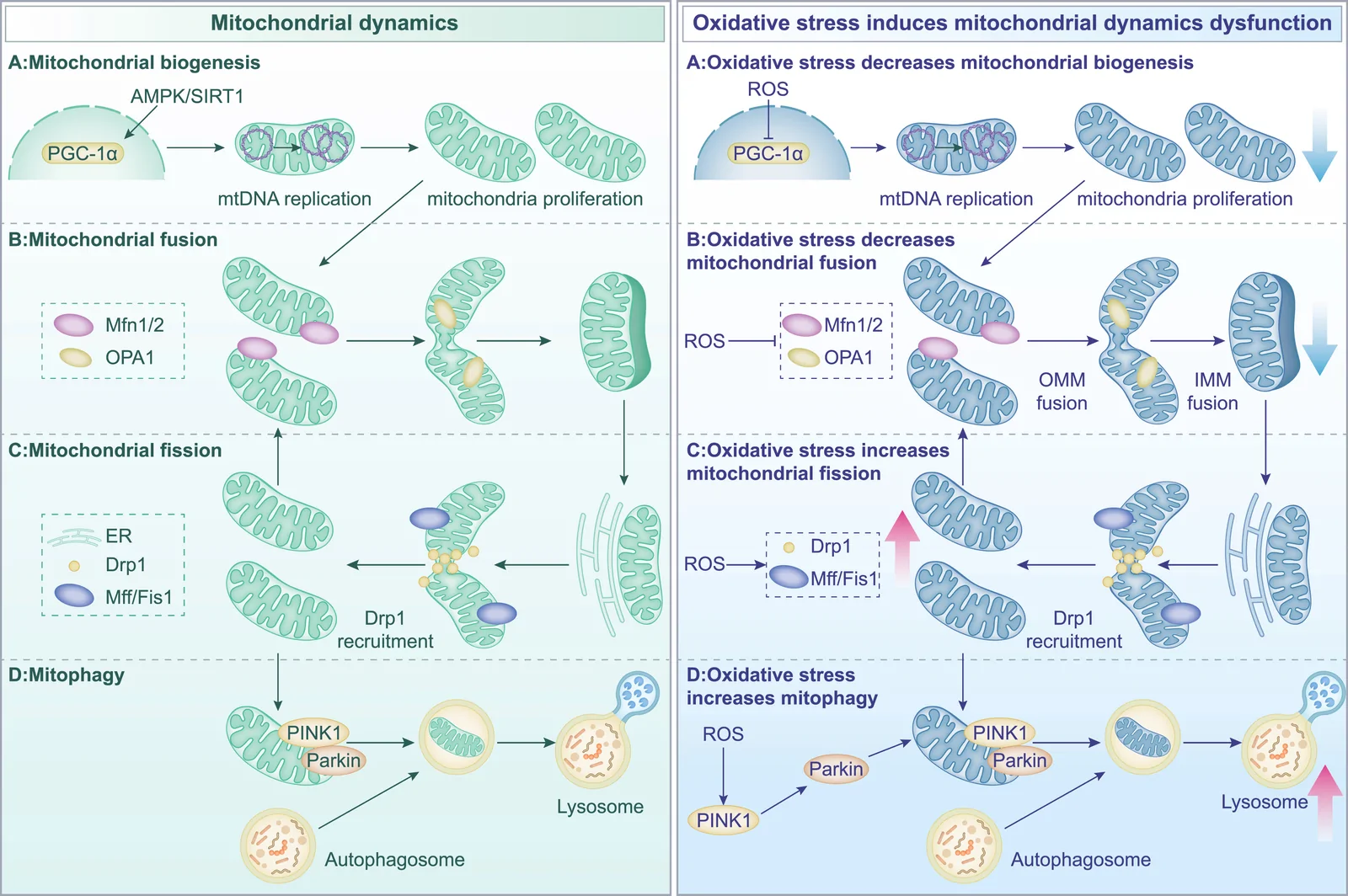
(1) Mitochondrial dynamics. (2) Oxidative stress induces mitochondrial dynamic dysfunction.

First, the mitochondrial respiratory chain generates energy, intertwining electron exchange among various complex components and facilitating proton transfer, thereby generating an electrochemical gradient crucial for ATP synthesis. In the ETC, electrons leak from complexes I, II, and III to reduce oxygen to produce superoxide (•O_2_−), which can be converted by SOD into hydrogen peroxide (H_2_O_2_). H_2_O_2_ can further generate •OH via the Fenton reaction; superoxide production from electron leakage represents the predominant source of mtROS [95]. Studies have identified two main ROS-producing sites in Complex I: flavin mononucleotide (FMN), which accepts electrons from NADH, and the Q-binding site [96]. ROS are also produced at complex III of the ETC [97]. ROS generated by mitochondria during OXPHOS are the principal intracellular source, whereas dysfunctional mitochondrial ROS may lead to oxidative damage [98].

Excessive accumulation of mitochondrial metabolites (such as succinate and NADH) exacerbates oxidative stress. Succinate, a pivotal intermediate in the tricarboxylic acid (TCA) cycle [99], can trigger reverse electron transfer (RET) when it accumulates abnormally in the mitochondrial matrix. During RET, electrons flow backward from complex II to complex I, resulting in massive production of superoxide anions (•O_2_−) at complex I and thereby inducing oxidative stress. Meanwhile, elevated levels of NADH—a key cellular reducing equivalent—increase the reduction state of upstream electron carriers in the electron transport chain, particularly complex I. When electron transport is impaired or slowed, electron leakage at complex I rises substantially, further promoting a burst of superoxide anions [100] and synergistically aggravating oxidative stress. Excessively high NADH also disrupts cellular redox homeostasis by causing over-reduction of the thioredoxin (Trx) system, which in turn inhibits the reactivation of peroxiredoxins (Prxs). This impairment compromises the cell’s ability to scavenge peroxides (e.g., H_2_O_2_) and exacerbates oxidative stress [101].

Under conditions of cellular stress, substantial quantities of Ca2+ are released from the endoplasmic reticulum lumen and translocate to mitochondria via mitochondria-associated ER membranes. Overloading of mitochondrial Ca2+ triggers the opening of the mitochondrial permeability transition pore, resulting in dissipation of the mitochondrial membrane potential and impaired electron transport chain activity. This cascade subsequently promotes reactive oxygen species production and amplifies oxidative stress within cells [70]. The endoplasmic reticulum additionally acts as a prominent endogenous source of reactive oxygen species. During ER stress, its own ROS production increases and can diffuse to adjacent mitochondria, damaging mitochondrial respiratory chain complexes [70]. This creates a positive feedback loop of “ROS-induced ROS release,” further amplifying oxidative damage.

Second, oxidative stress induces mitochondrial membrane potential depolarization, mitochondrial metabolism, and dysfunction of mitochondrial dynamics. Excessive ROS-induced oxidative stress induces mPTP opening and induces mitochondrial membrane potential depolarization (ΔΨ), stimulating mitochondrial impairment [102]. Increased ROS production damages mitochondrial DNA, protein, and lipid, eventually leading to mitochondrial dysfunction [103]. Consequently, a self-amplifying vicious cycle is established: mitochondrial dysfunction promotes increased ROS production, which further exacerbates mitochondrial impairment, leading to even greater ROS generation and a sustained escalation of oxidative stress.

Evidence from broader mitochondrial and non-OSAHS disease models suggests that oxidative stress induces mitochondrial metabolism dysfunction, which manifests as overproduction of ROS in mitochondria, reducing the TCA cycle and OXPHOS, decreasing ATP production, and leading to further overproduction of ROS [81]. Oxidative stress disrupts mitochondrial membrane lipid homeostasis, particularly impacting the balance of cardiolipin and cholesterol, which subsequently drives mitochondrial dysfunction. Specifically, ROS bursts directly target cardiolipin, a phospholipid enriched in polyunsaturated fatty acids within the inner mitochondrial membrane, initiating its lipid peroxidation [104]. Peroxidized cardiolipin compromises the structural integrity of respiratory chain supercomplexes, particularly complexes III and IV, leading to reduced electron transfer efficiency and increased electron leakage. This not only impairs oxidative phosphorylation but also further amplifies ROS production, establishing a self-perpetuating vicious cycle [105]. Concurrently, oxidative stress disrupts mitochondrial cholesterol metabolic homeostasis, potentially resulting in its abnormal accumulation within the inner membrane. This accumulation increases membrane rigidity, impairs the function of membrane proteins, and critically hinders the transport of endogenous antioxidants such as glutathione into the mitochondrial matrix, thereby severely weakening the organelle’s antioxidant defense capacity [106]. The two pathological pathways of cardiolipin peroxidation and cholesterol dysregulation are not independent; rather, they interact synergistically through shared nodes such as “glutathione depletion.” Cholesterol abnormalities exacerbate cardiolipin peroxidation by reducing antioxidant capacity, while cardiolipin damage further aggravates cholesterol accumulation and oxidative modification through increased ROS production. Oxidative stress further impairs mitochondrial membranes through triggering mitochondrial lipid peroxidation alongside the production of malondialdehyde (MDA) and 4-hydroxynonenal (4-HNE)—molecules capable of inflicting additional harm on proteins, lipids, and mtDNA [[107], [108], [109]]. Oxidation of cardiolipin induced by ROS attack compromises the efficiency of the mitochondrial respiratory chain [106]. Collectively, these findings indicate that oxidative stress induces and amplifies mitochondrial metabolic dysfunction, ultimately leading to mitochondrial bioenergetic failure, altered membrane permeability, and activation of apoptotic signaling [41].

Oxidative stress induces mitochondrial dynamic dysfunction, including fission, fusion, mitophagy, and mitochondrial biogenesis [110,111]. Enhanced levels of ROS upregulate Drp1, fission 1 (Fis1), and mitochondrial fission factor (Mff), leading to enhanced fission [112,113]. Similarly, increasing ROS levels reduce mitochondrial fusion through the downregulation of the Mfn1 and Mfn2 proteins, and OPA1 [114]. In addition, oxidized cardiolipin dissociates from the inner membrane and may translocate to the outer membrane, thereby impairing mitochondrial fusion [51]. The increase in mitochondrial fission may activate the PINK1/Parkin-dependent mitophagy pathway, and ROS may promote the recruitment of PINK1 and Parkin in the OMM [115]. However, with progression, the number of inefficient mitophagy cycles increases, resulting in the accumulation of damaged mitochondria. In addition, ROS overproduction induces the activation of redox-sensitive adenosine monophosphate (AMP)-activated protein kinase (AMPK), which may activate the PGC-1α promoter and increase mRNA levels, inducing deregulation of mitochondrial biogenesis and triggering mitochondrial mass decrease [116,117].

The above discussion summarizes the general mechanisms by which mitochondrial dysfunction and oxidative stress interact and mutually reinforce each other based on evidence from broad mitochondrial biology. Some of these mechanisms have been directly demonstrated in OSAHS/CIH models. For instance, excessive mtROS production, mPTP opening, mitochondrial membrane potential depolarization, increased mitochondrial fission, decreased mitochondrial fusion, PINK1/Parkin-dependent mitophagy, activation of the Nrf2 antioxidant pathway, and alterations in SOD activity and MDA levels have been observed in various CIH-exposed cell and animal models. However, other mechanisms discussed above—such as succinate-induced RET, cardiolipin peroxidation, mitochondrial cholesterol accumulation, glutathione depletion, and their proposed synergistic interactions—have not been directly validated in OSAHS/CIH models and are derived from the broader mitochondrial biology literature. In the following section, we review the specific mechanisms of mitochondrial dysfunction and oxidative stress that have been directly validated in OSAHS/CIH models and their associations with OSAHS-related complications.

## 3 Mitochondrial dysfunction and oxidative stress in OSAHS-associated complications

In OSAHS, CIH serves as the upstream trigger and central factor leading to mitochondrial dysfunction. The specific mechanisms involve: the repeated hypoxia/reoxygenation cycles characteristic of CIH directly impair the activity of ETC complexes, particularly Complexes I and III, increasing electron leakage; concurrently, CIH can induce abnormal opening of the mPTP, leading to depolarization of the mitochondrial membrane potential and potentially triggering mitophagy. These direct injuries subsequently result in reduced efficiency of ATP production and establish the primary source for excessive ROS generation. Critically, this CIH-triggered mitochondrial ROS overproduction creates a self-amplifying, vicious cycle [41,118]. The initial burst of ROS further exacerbates mitochondrial damage by inducing lipid peroxidation (notably of cardiolipin), protein oxidation, and mtDNA mutations, thereby deepening the respiratory chain dysfunction and creating even more ROS—a detrimental positive feedback loop [119]. This sustained oxidative stress propagates systemic inflammation. It activates redox-sensitive transcription factors (e.g., NF-κB) and the NLRP3 inflammasome, driving the release of pro-inflammatory cytokines (e.g., IL-6, TNF-α) and establishing a state of chronic, low-grade systemic inflammation. This inflammatory milieu, in turn, can feedback to impair mitochondrial function in various tissues (e.g., endothelium, cardiomyocytes, neurons), forming a second, larger pathogenic loop [120,121]. Importantly, after CIH initiates this process, the causal hierarchy between mitochondrial injury and oxidative stress becomes complex and self-amplifying, rather than a simple linear sequence. Ultimately, this integrated vicious cycle—where CIH induces mitochondrial ROS, which worsens mitochondrial dysfunction—which amplifies both local oxidative stress and systemic inflammation— which then further impairs mitochondria—serves as the core engine driving the progression of OSAHS and the development of its multi-system comorbidities, including cardiovascular, metabolic, and neurocognitive disorders. The subsequent sections will concentrate on reviewing the key functions of mitochondrial dysfunction and oxidative stress in OSAHS-associated complications (Fig. 4) .

**Fig. 4:**
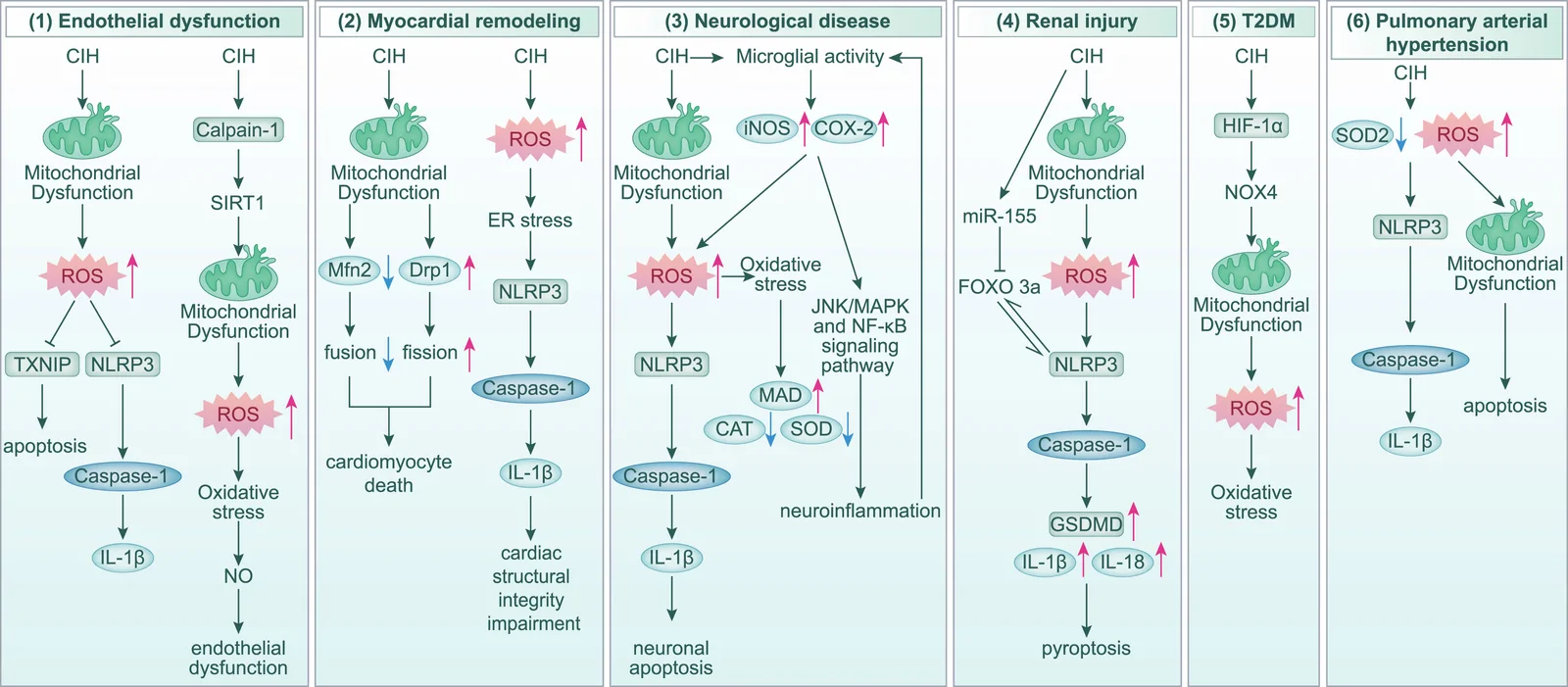
Mitochondrial dysfunction and oxidative stress in OSAHS-associated complications, including CVD (endothelial dysfunction and myocardial remodeling), neurological diseases, renal injury, type 2 diabetes mellitus, and lung diseases.

### 3.1 Cardiovascular diseases (CVD) in OSAHS

OSAHS-associated cardiovascular diseases include coronary artery disease, arrhythmias, cardiac hypertrophy, myocardial infarction, and heart failure [122]. Endothelial dysfunction and myocardial remodeling contribute largely to the development of CVD in patients with OSAHS.

#### 3.1.1 Mechanisms of mitochondrial dysfunction and oxidative stress in CIH-induced endothelial dysfunction

CIH-induced endothelial dysfunction includes impairment of endothelium-dependent vasodilation, increased arterial blood pressure and stiffness, and endothelial barrier dysfunction, which ultimately leads to vascular remodeling and atherosclerosis [123]. Depressed circulating levels of nitric oxide (NO) are the manifestation of endothelial dysfunction in patients with OSAHS [[124], [125], [126], [127]].

CIH-induced mitochondrial dysfunction and oxidative stress are key mechanisms causing endothelial dysfunction in OSAHS. CIH-induced mitochondrial dysfunction is characterized by changes in mitochondrial morphology, which involve loss of mitochondrial integrity, destruction of mitochondrial cristae, enlargement of the mitochondria (bleb-like), loss of mitochondrial membrane potential, opening of the mPTP, mtDNA damage, and excess production of mtROS in the aortas of mice and human coronary artery endothelial cells (HCAECs) [13,16,29,128]. Moreover, mitochondrial dynamic imbalance is induced by CIH, which is marked by decreased Mfn2 protein and increased Drp1 protein expression in HCAECs and mouse aortas [13,16,129]. Excessive mtROS cause oxidative stress, manifested as increased MDA levels, increased NOX2 and NOX4 levels, and decreased SOD and GSH levels in mouse aortas and HCAECs [13,16,70]. Furthermore, this mitochondrial bioenergetic failure and the resultant oxidative stress can reduce the levels of NO and form more reactive nitrogen species, ONOO-, which can oxidize lipids, proteins, DNA, and BH4, leading to increased ROS production. Consequently, vascular endothelial function is affected in OSAHS [13,[130], [131], [132]]. Additionally, mitochondrial dysfunction and oxidative stress induced by CIH may trigger endothelial apoptosis via activation of the TXNIP/NLRP3/IL-1β signaling pathway [13].

#### 3.1.2 Mechanisms of mitochondrial dysfunction and oxidative stress in CIH-induced myocardial remodeling

CIH-induced myocardial remodeling involves left ventricular (LV) hypertrophy, LV dilatation, increased interstitial fibrosis, and apoptosis [25,133]. Cardiac remodeling can cause contractile dysfunction, leading to an increased risk of arrhythmias and heart failure [134].

CIH directly impairs cardiac mitochondrial function by disrupting mitochondrial dynamics. Specifically, it regulates key dynamic proteins, lowering Mfn2 and boosting Drp1 expression, leading to an imbalance that promotes excessive mitochondrial fragmentation and subsequent cardiomyocyte death [129]. This dynamics imbalance is a critical facet of the overall dysfunction. Furthermore, CIH-induced mitochondrial bioenergetic impairment ultimately contributes to pathological cardiac remodeling, manifesting as left ventricular hypertrophy and contractile dysfunction [129]. CIH-induced oxidative stress includes increased ROS and MDA levels, increased NOX2 and NOX4 levels, and decreased SOD and GSH levels in mouse cardiomyocytes [24]. CIH-induced oxidative stress can activate ER stress and the NLRP3/caspase-1 signaling pathway, which may lead to cardiomyocyte pyroptosis, resulting in impaired cardiac structural integrity and a distinct reduction in ejection fraction and fractional shortening [24].

Overall, the activation of ROS and upregulation of mitochondrial dysfunction are key principles in ultimately increasing cardiovascular risk in patients.

### 3.2 Neurological disease in OSAHS

OSAHS-associated neurological diseases include neurocognitive and behavioral impairments, such as Alzheimer’s and Parkinson’s diseases [135]. Excessive CIH-induced neuroinflammatory responses may result in neuronal damage and central nervous system dysfunction in OSAHS. Oxidative stress and mitochondrial damage likely induce neurological impairments in OSAHS [136].

The roles of specific facets of mitochondrial dysfunction and oxidative stress in OSAHS-associated neurological diseases have been demonstrated. CIH increases mitochondrial membrane depolarization, augments mtROS release, and enhances the accumulation of damaged mitochondria. The NLRP3 inflammasome complex may be activated by mtROS and finally results in upregulation of neuronal apoptosis [45,137]. CIH-induced mitochondrial bioenergetic failure enhances microglial activation and aggravates neuroinflammation. CIH-induced ROS overproduction contributes to oxidative stress in mice microglia, manifested as decreasing levels of SOD and CAT expression and increasing levels of MDA [31,138]. CIH can also directly activate microglial cells, which express inducible nitric oxide synthase (iNOS) and COX-2 isoforms, leading to a further increase in ROS. Activated microglia drive the JNK/MAPK and NF-κB signaling pathways, contributing to the immunological inflammatory response with the production of cytokines including IL-1β, IL-6, and TNF-α [[139], [140], [141]]. Inflammatory cytokines further contribute to ROS production in the microglia, thereby promoting oxidative stress and aggravating neurological inflammatory injury [33,[142], [143], [144]].

### 3.3 Renal injury in OSAHS

OSAHS has been shown to contribute to advanced chronic kidney disease (CKD) via the direct impact of CIH on the kidney and activation of the systemic and renal renin-angiotensin systems [145,146]. Damage to the renal tubular epithelial cells is considered as the main inducer of CKD development. The mechanisms involved are complex, and mitochondrial bioenergetic impairment and sustained oxidative stress may play vital roles in the development of OSAHS-associated renal injury.

CIH promotes mitochondrial dysfunction and enhances renal injury. Specifically, mitochondrial depolarization and accumulated ROS may trigger assembly of the NLRP3 inflammasome and activation of caspase‐1 and IL‐1β release, leading to elevated apoptosis of renal tubular epithelial cells [35,147]. In addition, CIH causes mitochondrial dynamic imbalance in renal tissues, which is manifested by increased expression of Fis1 and decreased expression of Mfn1, resulting in renal injury [148,149].

Moreover, CIH activates oxidative stress in renal tissue, as evidenced by increased MDA levels and decreased SOD, CAT, and GSH activities. CIH induces oxidative stress leading to pyroptosis via the TLR-4/NF-κB/NLRP3/caspase-1 pathway, which results in renal tubular epithelial cell injury, ultimately leading to a thicker glomerular basement membrane and mesangium, as well as infiltration of inflammatory cells [34].

### 3.4 Type 2 diabetes mellitus (T2DM) in OSAHS

CIH is independently associated with insulin resistance, and OSAHS contributes to T2DM [150]. CIH may activate β-cell dysfunction, manifested as increasing basal insulin secretion and impaired glucose-stimulated insulin secretion [151,152].

Insulin resistance is associated with CIH-induced oxidative stress, mitochondrial bioenergetic compromise, and inflammatory activation [153]. CIH upregulates HIF-1α in pancreatic β cells, promoting mtROS generation, leading to elevated basal insulin secretion and insulin resistance [154]. Wang et al. further demonstrated that HIF-1α transcriptionally activates NOX4 in pancreatic β cells, increasing mtROS/H_2_O_2_ generation, and that these effects are abolished in HIF-1α-heterozygous mice and β-cell-specific HIF-1α knockout mice [36]. Concurrently, a study demonstrated that CIH-induced oxidative stress in the pancreas activates TRB3 and phosphorylated JNK (p-JNK), leading to upregulation of p-IRS-1Ser307 and downregulation of p-AKTSer473, thereby impairing the IRS-1/AKT insulin signaling pathway and resulting in insulin resistance [155]. Current reviews further elucidate that OSAHS-induced mitochondrial dysfunction and elevated ROS production activate the NLRP3 inflammasome, leading to increased levels of pro-inflammatory cytokines such as TNF-α, IL-6, and IL-1β, which ultimately interfere with insulin signaling and contribute to insulin resistance [156].

In summary, OSAHS-associated diabetes can be induced by mitochondrial dysfunction and oxidative stress; the specific pathways involved, however, remain largely unexplored and warrant further investigation in future studies.

### 3.5 Lung diseases in OSAHS

OSAHS-associated lung diseases include chronic obstructive pulmonary disease (COPD), pulmonary arterial hypertension (PAH), asthma, and interstitial lung diseases [157,158]. Mitochondrial dysfunction, oxidative stress, and related inflammation play vital roles in cellular development [159,160].

Mice exposed to CIH exhibit increased ROS production, diminished mitochondrial membrane potential, and impaired mitochondrial respiration in lung tissue [161,162]. CIH also reduces SOD2 protein levels, which compromises the mitochondrial antioxidant defense capacity. Moreover, CIH-induced mtROS activates the NLRP3 inflammasome, thereby promoting apoptosis of human pulmonary artery smooth muscle cells, eventually leading to pulmonary inflammation and deteriorating vascular remodeling [15,159]. CIH induces robust pulmonary inflammation and oxidative stress, primarily mediated through the activation of the NF-κB signaling cascade, which drives the inflammatory response. Concurrently, CIH triggers a compensatory upregulation of the Nrf2/HO-1 pathway as a protective mechanism to mitigate oxidative stress [17].

However, based on limited studies, mitochondrial damage, oxidative stress, the NLRP3 inflammasome, and related inflammation and immunoreaction are likely vital to lung diseases in patients with OSAHS and may be a therapeutic target; thus, the precise mechanisms underlying these pathologies must be further elucidated.

## 4 Potential targeted therapeutic strategies for mitochondrial dysfunction and oxidative stress in OSAHS

### 4.1 Mitochondrial ROS scavengers

#### 4.1.1 Mito-TEMPO and Mito-TEMPOL

Mito-TEMPO and Mito-TEMPOL are two structurally related mitochondria-targeted superoxide dismutase mimetics that have demonstrated direct protective effects in multiple OSAHS/CIH preclinical models by scavenging mitochondrial superoxide, though they remain in the preclinical stage of development (Table 1, Fig. 5).

**Table 1:** Potential therapeutic drugs targeting mitochondrial dysfunction and oxidative stress for OSAHS-associated complications.

Name	Category	Agents	Mechanisms	Primary Efficacy	Reference	Model/evidence source	Clinical evidence in OSAHS	Main limitation
Mito-TEMPO	Nitroxide	Synthetic compound	Inhibiting ROS production; Suppressing the activation of the NLRP3 inflammasome; Inhibiting the opening of the mPTPs; Ameliorating the blebbing of mitochondria	Attenuates vascular endothelial dysfunction (CIH rat and cell model); Attenuates cognitive dysfunction (CIH cell model); Attenuates pulmonary vascular remodeling (CIH mouse model)	[13] [14] [15]	CIH rat model and CIH cell model (HCAECs) (cardiovascular); CIH cell model (BV-2 microglial cell) (neurological); CIH mouse model (lung)	No	Lack of detailed in vivo behavioral analyses and insufficient pharmacokinetic data; No long-term safety or combination therapy studies
Mito-TEMPOL	Nitroxide	Synthetic compound	Inhibiting ROS production; Inhibiting the opening of the mPTPs; Ameliorating the blebbing of mitochondria	Attenuates vascular endothelial dysfunction	[16]	CIH mouse model (aorta); CIH cell model (HCAECs)	No	Lack of detailed in vivo behavioral analyses and insufficient pharmacokinetic data; No long-term safety or combination therapy studies
Tempol	Nitroxide	Synthetic compound	Suppressing the NF-κB inflammatory pathway; Activating the Nrf2/HO-1 antioxidant signaling pathway; Ameliorating oxidative stress; Suppressing the TRB3/p-JNK pathway; Restoring IRS-1/AKT signaling	Attenuates pulmonary vascular remodeling and lung injury (CIH rat and mouse model); Attenuates insulin resistance (CIH rat and mouse model)	[17] [18] [19] [20] [21]	CIH rat and mouse model (lung, pancreas)	No	Absence of OSAHS-specific human trials; Pharmacokinetic challenges
N-acetylcysteine (NAC)	Amino acid l-cysteine	Old drug (repurposed)	Inhibiting ROS production; Reversing the increasing level of MDA; Upregulating mitochondrial membrane potential; Upregulating the level of p-eNOS, NO	Attenuates vascular endothelial dysfunction	[16]	CIH mouse model (aorta); CIH cell model (HCAECs)	No	Clinical evidence in OSAHS patients is lacking. Long-term efficacy and safety in OSAHS-associated complications remain to be established
Ginsenoside Rg1	Triterpenoid saponins	Natural products	Inhibiting ROS production; Restoring mitochondrial membrane potential; Inhibiting the Calpain-1/PP2A signaling pathway; Promoting eNOS phosphorylation and NO production	Attenuates vascular endothelial dysfunction	[16]	CIH mouse model (aorta); CIH cell model (HCAECs)	No	Clinical evidence in OSAHS patients is lacking. Long-term efficacy and safety in OSAHS-associated complications remain to be established
Pinocembrin	Flavonoid	Natural products	Inhibiting NLRP3 inflammasome activation; Enhancing BNIP3-mediated mitophagy; Inhibiting the mtROS-NLRP3 axis and inflammatory cytokine production; Inhibiting mitochondrial damage and cell apoptosis	Inhibits neuroinflammation and attenuates cognition dysfunction	[22]	CIH mouse model (brain)	No	Other aspects of direct mitochondrial effects (membrane potential, respiratory chain, dynamics) not fully characterized in OSAHS models
Rapamycin	Macrolide	Natural products	Inhibiting mTOR; Upregulating autophagy; Inhibiting NLRP3 inflammasome activation; Reducing mRNA levels of IL-1β and IL-18; Inhibiting cell pyroptosis	Attenuates renal injury	[23]	CIH mouse model (kidney)	No	Mitochondrial protective effects have not been investigated in CIH models
Resveratrol (RV)	Stilbenoid	Natural products	Inhibiting ROS production; Activating AMPK pathway; Inhibiting the mTOR pathway; Blocking activation of NLRP3 inflammasome; Inhibiting pyroptosis; Upregulating Sirt1 and activating the PI3K/AKT signaling pathway	Protects myocardial structure and function (CIH rat model); Attenuates insulin resistance (CIH rat model)	[24] [25] [26]	CIH rat model (heart, blood and liver)	No	Only in vivo data from a CIH rat model are available; CIH in vitro experiments and clinical studies in OSAHS patients are lacking
Sulforaphane (SFN)	Phytocompound	Natural products	Reducing caspase 3-dependent cell apoptosis; Inhibiting CIH-induced oxidative stress; Enhancing Nrf2 nuclear translocation; Increasing autophagy	Attenuates cognition dysfunction	[27]	CIH mouse model (hippocampal neurons)	No	In vitro experiments in CIH models are lacking. Direct mitochondrial endpoints (membrane potential, mtROS, respiratory chain efficiency, mitochondrial dynamics) were not measured
Honokiol (HNK)	Lignan	Natural products	Inhibiting ROS production; Activating Nrf2/ARE pathway; Inhibiting cell apoptosis; Ameliorating oxidative stress	Preserves pancreatic β cell function	[28]	CIH rat model (pancreas); CIH cell model (INS-1 cells)	No	Mitochondrial dynamics, respiratory function, and quality control effects not yet elucidated in OSAHS models
Astragaloside IV	Triterpenoid saponin	Natural products	Inhibiting ROS production; Abolishing the upregulated inflammatory factors secretion; Restoring mitochondrial membrane potential; Downregulating the level of calpain-1; Activating the SIRT1/AMPK pathway; Promoting eNOS phosphorylation and NO production	Attenuates vascular endothelial dysfunction	[29]	CIH cell model (HCAECs); CIH mouse model (aorta)	No	No clinical trials, limited to cellular and animal experiments
Betulin	Triterpenoid	Natural products	Ameliorating SREBP2 expression and rescuing ISCU1/2 levels in CIH mouse model; Partially restoring mitochondrial complex I activity in OSAHS serum-treated HUVECs; Ameliorating systolic blood pressure elevation	Attenuates vascular endothelial dysfunction	[30]	CIH mouse model (vascular endothelial cells); HUVECs with OSA serum	No	Direct in vivo evidence for betulin-mediated mitochondrial protection (membrane potential, mtROS, respiratory chain) in CIH cardiovascular models is still lacking
TD139	Thiodigalactoside	Synthetic compound	Inhibiting Galectin-3/NLRP3 axis; Reducing LPS-induced neuroinflammation and oxidative stress in HMC3 microglial cells	Inhibits neuroinflammation	[31]	OSAHS patient cohort (clinical); LPS-stimulated HMC3 cell model (in vitro)	Observational (target-level)	No interventional clinical data for TD139 in OSAHS patients. Direct mitochondrial protective effects and in vivo efficacy in CIH models have not been investigated
miR-146a-5p	microRNA	miRNA	Inhibiting the activation of NLRP3 inflammasome; Inhibiting HIF1α; Down-regulating mtROS level; Reducing mRNA levels of IL-1β and IL-18	Inhibits neuroinflammation and attenuates cognition dysfunction	[32]	CIH mouse model (brain); CIH cell model (microglia)	No	Direct mitochondrial protective evidence in CIH models have not been investigated
Pseudoginsenoside GQ	Saponin	Synthetic compound	Inhibiting ROS production; Reducing level of IL-1β; Inhibiting HMGB1/TLR4/NF-κB pathway activation; Ameliorating CIH-induced cell apoptosis	Inhibits neuroinflammation and attenuates cognitive impairment	[33]	CIH mouse model (brain); CIH cell model (microglia)	No	Direct mitochondrial protective effects (membrane potential, respiratory chain efficiency, mitochondrial dynamics) have not been investigated in CIH models. Clinical evidence in OSAHS patients is lacking
MCC950	Diarylsulfonylurea	Synthetic compound	Inhibiting ROS production; Inhibiting NLRP3 inflammasome activation; Reducing the production of IL-1β and IL-18	Attenuates renal injury	[34]	CIH cell model (HK-2, TCMK-1); CIH rat model (kidney)	No	Direct mitochondrial protective effects (membrane potential, respiratory chain efficiency, mitochondrial dynamics) have not been investigated in CIH models
anti-miR-155	microRNA	miRNA	Inhibiting miR-155; Inhibiting ROS production; Inhibiting NLRP3 inflammasome activation; Reducing level of IL-1β; Ameliorating CIH-induced cell apoptosis	Attenuates renal injury	[35]	CIH mouse model (kidney); CIH cell model (HK-2)	No	Direct mitochondrial effects (bioenergetics, membrane integrity, quality control) have not been investigated in CIH models
Digoxin	Cardiac glycoside	Old drug (repurposed)	Inhibiting HIF-1α expression; Inhibiting ROS production; Inhibiting NOX4	Suppresses HIF-1α expression and diminishes NOX4-derived ROS production in pancreatic β-cells in CIH mouse model; No clear evidence supports efficacy in alleviating T2D/IR	[36]	CIH mouse model (pancreas, β-cell)	No	Mitochondrial effects have not been defined in CIH models; No clear evidence supporting efficacy in alleviating T2D/IR
Melatonin	Indoleamine	Natural products	Inhibiting ROS production; Increasing nitric oxide (NO) bioavailability	Attenuates insulin resistance	[37]	CIH hamster model (metabolic, microvascular)	No	Dosing and timing concerns for glucose homeostasis; In vitro experiments in CIH models are lacking
Dapagliflozin	SGLT2 inhibitors	Synthetic compound	Improving sleep parameters (e.g., Apnea-Hypopnea Index, oxygen desaturation index); Benefiting metabolic parameters, nocturnal heart rates, and heart rate variability	Attenuates vascular endothelial dysfunction; Attenuates insulin resistance	[38] [39] [40]	OSAHS patient RCT (clinical)	Interventional	In vivo and in vitro evidence in CIH/OSAHS models is lacking

**Fig. 5:**
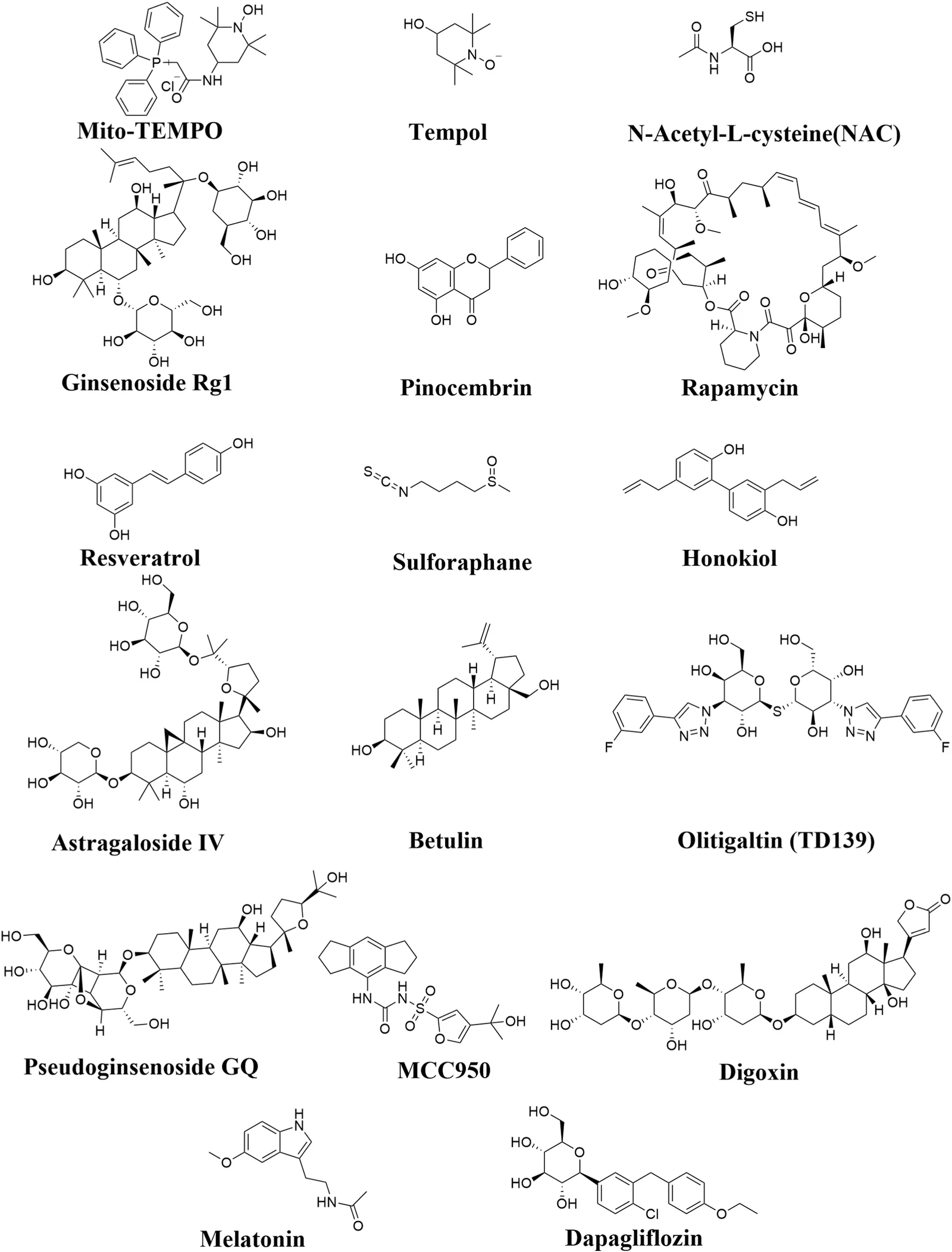
Structures of drugs targeting mitochondrial dysfunction and oxidative stress for OSAHS-associated complications.

Mito-TEMPO and Mito-TEMPOL achieve targeted mitochondrial accumulation through mitochondrion-specific molecular design. As a SOD mimetic, it precisely eliminates excess superoxide radicals within mitochondria, thereby attenuating mitochondrial oxidative stress and preserving mitochondrial function [163]. Mito-TEMPOL, a structural derivative of Mito-TEMPO, incorporates a hydroxyl group (-OH) at the 4-position of the TEMPO moiety [164]. This modification significantly enhances its ROS scavenging capacity and redox cycling properties, which may contribute to more potent mitochondrial antioxidant efficacy under specific conditions [165]. Direct evidence from CIH models shows their efficacy across organ systems.

In cardiovascular injury models, Mito-TEMPO significantly reduced mtROS and prevented the opening of mPTP in a CIH rat model. This intervention helped preserve mitochondrial integrity and decreased endothelial cell apoptosis by modulating the TXNIP/NLRP3/IL-1β signaling pathway, ultimately playing a role in the progression of CVD [13]. Mito-TEMPOL treatment improved cell viability, reduced intracellular ROS levels, reversed the downregulation of mitochondrial membrane potential in HCAECs, and protected against the impairment of endothelial relaxation in the aortas of mice in another CIH animal model [16].

In neurological injury models, Mito-TEMPO application significantly suppressed the activation of the TXNIP/NLRP3/GSDMD pyroptosis pathway in BV-2 microglial cells, thereby reducing neuroinflammation and neuronal injury; this established a clear signaling axis of “CIH → mtROS → TXNIP/NLRP3/GSDMD → cognitive impairment” that can be interrupted at the ROS node [14]. Supporting indirect evidence from related comorbidities shows efficacy in models of vascular dementia and diabetes-associated cognitive decline, where Mito-TEMPO improved mitochondrial bioenergetics, reduced tau oligomerization, and preserved blood-brain barrier integrity by reducing pericyte loss [166,167].

In lung injury models, Mito-TEMPO supplementation reverses CIH-induced mitochondrial bioenergetic impairment, redox imbalance, and NLRP3 inflammasome activation [15]. Indirect evidence supporting its potential application in OSAHS-related lung disease stems from the role of its target, SOD2. The dysregulation of SOD2 is a known contributor to the development of pulmonary inflammation and pulmonary arterial hypertension. In experimental models, reduced SOD2 activity is correlated with increased ROS levels and upregulated NLRP3 inflammasome activity [159,168].

Despite promising multi-system preclinical data, clinical translation is at an early stage. Key limitations include a lack of detailed in vivo behavioral analyses in OSAHS models, insufficient pharmacokinetic data, and no long-term safety or combination therapy (e.g., with CPAP) studies.

#### 4.1.2 Tempol

Tempol, a cell membrane-permeable, broad-spectrum SOD mimetic, has shown direct efficacy in OSAHS models of pulmonary and metabolic injury, but remains in preclinical development for these indications.

Tempol is a cell membrane-permeable SOD mimetic and an effective free radical scavenger. The fundamental distinction between Tempol and Mito-TEMPO lies in their specificity: Tempol functions as a broad-spectrum cellular antioxidant, whereas Mito-TEMPO achieves specific targeting and accumulation within mitochondria through chemical modification. Compared to Mito-TEMPOL, TEMPOL lacks mitochondrial targeting. In the OSAHS rat model, Tempol mitigates CIH-induced lung injury by suppressing the NF-κB inflammatory pathway and concurrently activating the Nrf2/HO-1 antioxidant signaling pathway. This dual mechanism attenuates oxidative stress and inflammatory responses, thereby exerting protective effects on pulmonary tissue [17]. Another CIH rat model study proved that tempol alleviates lung injury by regulating the miR-145-5p/Nrf2 axis and inhibiting autophagy [18]. Direct evidence from multiple CIH mouse models further confirms that Tempol alleviates pulmonary oxidative stress, inflammation, and fibrosis through additional modulation of the TLR4/Nrf2/GSH and miR-212-5p/SIRT6/HIF-1α signaling axes, with recent studies highlighting its novel role in suppressing ferroptosis [19,20].

In models of pancreatic injury and insulin resistance, direct evidence from CIH rat models shows that Tempol (100 mg/kg/d) attenuates islet cell apoptosis and insulin resistance by suppressing the TRB3/p-JNK pathway and oxidative stress markers (8-OHdG), while restoring IRS-1/AKT signaling [155]. Complementary studies confirm it reduces CIH-induced hyperglycemia and pancreatic inflammation via MAPK/NF-κB inhibition [21]. Indirect evidence from non-OSAHS models (e.g., cardiovascular ischemia-reperfusion) supports its broad antioxidant benefit in metabolic stress contexts, but human data in diabetic OSAHS patients are absent [169].

Clinically, Tempol is not yet developed for OSAHS, but it has been investigated for non-OSAHS indications [170]. Key limitations include the absence of OSAHS-specific human trials and pharmacokinetic challenges.

#### 4.1.3 N-acetylcysteine (NAC)

NAC, a glutathione precursor, has shown direct protective effects in preclinical models of OSAHS-related cardiovascular injury.

NAC has shown potential for application in the treatment and prevention of CVD due to its antioxidant and anti-inflammatory properties, primarily through mitigating oxidative stress, suppressing inflammation, and improving endothelial function [171,172]. Despite demonstrating promising outcomes in various in vitro and animal models, the definitive clinical efficacy of NAC in CVD therapy remains inconsistent, particularly as evidenced by mixed results from large-scale, high-quality randomized controlled trials. Regarding its use in OSAHS-related CVD, research involving NAC is currently limited to cellular and animal experimental levels. The findings showed that NAC therapy protects the aortas of mice from endothelium-dependent damage caused by CIH. Furthermore, NAC treatment significantly enhanced cell viability, elevated the levels of p-eNOS, NO, Mfn2 protein, as well as restored mitochondrial membrane potential, while simultaneously reducing the levels of mtROS, MDA, and the expression of Drp1 in CIH-exposed HCAECs, similar to the effects of ginsenoside Rg1 [16]. NAC exhibits cardioprotective effects in chronically hypoxic rats by lowering ROS levels and enhancing cardiac endothelial NO synthase activity and expression.

### 4.2 Modulators of mitochondrial dynamics and mitophagy

#### 4.2.1 Ginsenoside Rg1

Ginsenoside Rg1 is a natural chemical isolated predominantly from the traditional Chinese herb ginseng. It has been reported to have many therapeutic benefits on cardiovascular illnesses, including anti-atherosclerosis, anti-myocardial hypertrophy, and antivascular remodeling [[173], [174], [175]]. Research has demonstrated that ginsenoside Rg1 lessened the impact of CIH on VED through the eNOS/NO signaling mechanism, and it helped mitigate the mitochondrial dysfunction and oxidative stress triggered by CIH. Furthermore, Rg1 treatment was effective in preventing the decline in mitochondrial membrane potential in HCAECs and lowering the elevated mitochondrial ROS levels in both the aorta and HCAECs during CIH. These benefits were facilitated by the calpain-1/PP2A/eNOS signaling sequence. Ginsenoside Rg1 therapy also led to an increase in Mfn2 levels, a decrease in Drp1 levels—both key regulators of mitochondrial dynamics—and it reversed the heightened oxidative stress marker MDA both inside and outside of the cell [16].

#### 4.2.2 Pinocembrin (5,7-dihydroxyflavone)

Pinocembrin, which enhances mitophagic flux, has shown direct protective effects in models of OSAHS-related neuroinflammation and cognitive impairment.

Pinocembrin, also known as 5,7-dihydroxyflavone, is a bioflavonoid found in a wide range of botanicals, fungi, and even bee products, such as honey and propolis. This compound boasts a variety of medicinal properties, ranging from anti-inflammatory and antioxidant benefits to its roles in neuroprotection, antimicrobial, anticancer, and heart-healthy effects [176]. One study demonstrated that pinocembrin improved BNIP3-dependent mitophagy to regulate increased mtROS generation and NLRP3 inflammasome accumulation in brain impairment, leading to the regulation of NLRP3-mediated inflammation and inhibition of cell apoptosis during CIH [22]. Overall, pinocembrin has been shown to be a potential therapeutic drug for OSAHS-associated neuroinflammation and cognitive impairment, as well as to alleviate behavior and cognitive deficiencies in a CIH mouse model. This indicates that pinocembrin alleviates cytotoxicity by decreasing cell apoptosis and the accumulation of NLRP3 inflammasomes in CIH-treated microglia in vitro. By improving mitophagic flux, pinocembrin may help restore mitochondrial quality control, indirectly supporting mitochondrial bioenergetic function and redox homeostasis in the context of CIH-induced neuronal stress. Further investigation is warranted to determine whether pinocembrin also directly influences other aspects of mitochondrial integrity, such as membrane potential stability, respiratory chain efficiency, or mitochondrial dynamics.

#### 4.2.3 Rapamycin

Rapamycin, a key regulator of autophagy, has demonstrated direct protective effects in a model of OSAHS-related renal injury, potentially via modulating mitophagy.

Rapamycin serves as a key regulator of autophagy and impacts multiple cellular processes, such as protein production, mitochondrial energy generation, ribosome assembly, and cellular development. By inhibiting the mTOR signaling pathway, it not only modulates these functions but also affects the advancement of OSA [177]. In a CIH mouse model, a three-week course of rapamycin therapy protected mice from renal damage by inhibiting the mTOR/NLRP3 axis and upregulating autophagy. The rapamycin-treated group exhibited increased LC3 and Beclin 1 protein expression, reduced P62 levels, and decreased protein and mRNA levels of NLRP3, GMDSD, IL-18, IL-1β, and cleaved-caspase-1, thereby upregulating autophagy to reduce oxidative stress [23]. Given the interplay between autophagy, mitochondrial quality control, and inflammasome activation, rapamycin’s modulation of mTOR may also contribute to preserving mitochondrial function—such as supporting mitophagic clearance, maintaining membrane potential, and reducing ROS-mediated damage—in OSAHS-related organ injury. Further studies are warranted to delineate its direct effects on mitochondrial bioenergetics, dynamics, and redox homeostasis in the context of CIH.

### 4.3 Nrf2 pathway activators

#### 4.3.1 Resveratrol (RV)

Resveratrol, a classic Nrf2 pathway activator, has demonstrated direct protective effects in preclinical models of OSAHS-related cardiovascular and metabolic injury.

Resveratrol, a prevalent natural polyphenolic compound, is found in diverse plant species—most notably grapes, peanuts, and berry fruits. RV is known for its health benefits, such as antioxidative, cardioprotective, antitumor, insulin-sensitizing, neuroprotective, and anti-inflammatory effects [178]. In CVD, RV has attracted great attention from medical professionals for its effective roles in mitochondrial bioenergetic impairment, oxidative stress, inflammation, and apoptosis, and is regarded as a potent antioxidant capable of effectively suppressing ROS generation. RV displays favorable cardioprotective properties against cardiovascular dysfunction driven by CIH. Evidence indicates that RV triggers Nrf2 activation and upregulates SOD2 as well as heme oxygenase-1 (HO-1), which constitute the core pathway for relieving oxidative stress and restraining inflammasome formation, ultimately relieving myocardial injury induced by CIH. Following 5 weeks of CIH exposure, the RV-treated group showed fewer TUNEL-positive cells and reduced plasma concentrations of CK and LDH, protecting cardiovascular cells from pyroptosis. Resveratrol also reduced the production of MDA evoked by CIH, ameliorated the expression of CIH-induced NOX2 and NOX4, and restored cardiac antioxidant activity. RV increased AMPK activation and inhibited the mTOR signaling pathway, contributing to a decrease in NLRP3 production [24]. Another rat model study on CIH demonstrated that RV ameliorates CIH-induced cardiac remodeling by inhibiting the PI3K/AKT/mTOR signaling pathway. This inhibition restores autophagy activity, which in turn alleviates oxidative stress, suppresses cardiomyocyte apoptosis, and ultimately improves cardiac structure and function [25].

In models of metabolic dysfunction, RV exhibits beneficial effects on T2DM primarily through its potent antioxidant and anti-inflammatory properties. In a CIH rat model, RV ameliorates oxidative stress, improves insulin sensitivity, protects liver function, and modulates metabolism by upregulating SIRT1 expression and activating the PI3K/AKT pathway, providing direct evidence of its protective effects in the context of OSAHS-related metabolic dysfunction [26]. This aligns with and is supported by a broader body of indirect evidence from non-OSAHS models of T2DM, where RV exhibits beneficial effects primarily through its potent antioxidant and anti-inflammatory properties [179,180]. However, research specifically focused on the direct therapeutic application of RV for OSAHS-associated T2DM remains limited, though current animal studies have shown promising outcomes. Prior to its potential translation into clinical trials for OSAHS-related T2DM, further in-depth investigation into the underlying molecular mechanisms and associated signaling pathways is warranted.

#### 4.3.2 Sulforaphane (SFN)

Sulforaphane, another potent Nrf2 activator, has shown direct protective effects in an in vitro model of OSAHS-related neurocognitive dysfunction.

Sulforaphane (SFN), a phytocompound naturally isolated from the sprouts of numerous cruciferous vegetables, possesses antioxidant, anti-apoptotic, and anti-inflammatory activities [181]. Sulforaphane is utilized to treat neurodegenerative illnesses because of its ability to pass the blood-brain barrier [182] and has been shown to benefit patients with T2DM and CVD [181,183]. In an in vivo model, SFN enhanced Nrf2 nuclear translocation to promote antioxidant effects and activate autophagy, thereby reducing hippocampal neuronal apoptosis and ameliorating OSAHS-related neurocognitive dysfunction [27]. Importantly, by modulating the Nrf2 pathway, SFN may also help restore mitochondrial redox balance and support cellular quality control mechanisms, which are often impaired under conditions of chronic intermittent hypoxia. Given these multimodal mechanisms, SFN represents a promising neuroprotective candidate for the treatment of neurocognitive dysfunction in patients with OSAHS. However, its specific impact on mitochondrial bioenergetics, dynamics, and mitophagy in the context of CIH-related neuronal injury warrants further investigation in vitro and in clinically relevant models.

#### 4.3.3 Honokiol (HNK)

Honokiol, which activates the Nrf2/ARE pathway, has demonstrated direct protective effects in a model of OSAHS-related pancreatic injury and metabolic dysregulation.

Honokiol (HNK), a bioactive substance extracted from the bark of *Magnolia officinalis*, has long been utilized in traditional Chinese medicine practices. Modern research confirms this natural substance possesses an impressive pharmacological profile, demonstrating potent antioxidant, anti-inflammatory, and anticancer properties [184]. Findings in vivo and in vitro indicate that honokiol reduces ROS levels and attenuates oxidative stress through the Nrf2/ARE pathway, further improving the functioning of pancreatic β-cells in T2DM rats during CIH treatment. The results revealed that honokiol suppresses oxidative stress by reducing antioxidant enzymes and oxidation products in CIH rats. Furthermore, data indicate that honokiol defends INS-1 cells against HG or CIH + HG-induced apoptosis [28]. Given the pivotal role of mitochondrial redox homeostasis and metabolic stability in β-cell physiology, honokiol’s activation of the Nrf2 pathway may contribute to the maintenance of mitochondrial structural integrity, enhancement of bioenergetic performance, and reduction of oxidative injury in OSAHS-related metabolic dysregulation. Consequently, honokiol emerges as a promising therapeutic agent for OSAHS complicated by diabetes mellitus. Nevertheless, additional investigations are required to elucidate its specific impacts on mitochondrial dynamics, respiratory function, and quality control pathways within relevant pathological models.

### 4.4 Other multi-targeted agents and emerging strategies

#### 4.4.1 Astragaloside IV (As-IV)

Astragaloside IV, a multi-target natural product, has demonstrated direct protective effects in models of OSAHS-related cardiovascular injury, primarily by improving mitochondrial function via the calpain-1/SIRT1/AMPK axis.

Astragaloside IV (As-IV), a naturally occurring substance derived from *Astragalus membranaceus*, has a variety of pharmacological actions in cardiovascular disorders, including immune-modulatory, anti-inflammatory, antioxidant, and antifibrotic properties [[185], [186], [187]]. Although AS-IV has demonstrated significant protective effects in various models of cardiovascular diseases, direct clinical studies evaluating its efficacy for treating OSAHS-related cardiovascular diseases are currently scarce, with research predominantly confined to cellular and animal experiments. Among these, investigations that specifically focus on its modulatory effects on mitochondrial bioenergetics and redox homeostasis are relatively scarce. In human coronary artery endothelial cells (HCAECs) exposed to CIH, treatment with As-IV significantly reduced mtROS levels and restored the CIH-induced decline in mitochondrial membrane potential, thereby preserving mitochondrial integrity. In both in vivo (mouse serum and aorta) and in vitro (HCAEC supernatant) experiments, As-IV administration decreased levels of MDA and intracellular total ROS, while it enhanced the activity of SOD and the levels of GSH-Px. Consequently, it alleviated oxidative stress and improved nitric oxide (NO) bioavailability. The study demonstrates that As-IV exerts its endothelial-protective effects against OSAHS-associated cardiovascular complications by activating the calpain-1/SIRT1/AMPK signaling axis. This action fundamentally improves mitochondrial function and attenuates oxidative stress, thereby safeguarding vascular endothelial function [29]. Future work will require more in-depth basic research to further elucidate AS-IV’s specific molecular targets and signaling pathways in OSAHS models, as well as additional clinical trials to validate its therapeutic effectiveness and safety profile.

#### 4.4.2 Betulin

Betulin, a selective SREBP2 inhibitor, has the potential to improve mitochondrial bioenergetic function by modulating cholesterol metabolism and iron-sulfur cluster assembly, based on indirect evidence and pathway analysis; however, direct in vivo evidence for betulin-mediated mitochondrial protection in CIH-induced cardiovascular injury models is still lacking.

Betulin (B) and betulinic acid (BA) are naturally occurring pentacyclic triterpenoids with a lupane-type structure, renowned for their versatile therapeutic potential in combating infectious diseases and metabolic, neurological, and cardiovascular disorders [188]. Intermittent hypoxia triggers sterol regulatory element-binding protein 2 (SREBP2), a key transcription factor, which attaches to the promoter region of miR-210. This leads to inhibition of iron-sulfur cluster assembly enzymes, ultimately causing mitochondrial respiratory chain dysfunction [30,189]. Furthermore, SREBP2 indirectly affects mitochondrial function by regulating cholesterol synthesis and cellular uptake, leading to outcomes such as mitochondrial cholesterol accumulation, increased oxidative stress, and disturbances in mitochondrial dynamics [190]. These alterations collectively drive the development and progression of cardiovascular complications, including atherosclerosis and diabetic cardiomyopathy [191].

Evidence from both in vitro endothelial cell models and in vivo CIH mouse models has demonstrated that betulin, as a selective SREBP2 inhibitor, attenuates SREBP2 expression, rescues ISCU1/2 levels, partially restores mitochondrial respiratory complex I activity in OSAHS serum-treated endothelial cells, and alleviates IH-induced systolic blood pressure elevation [30]. Studies have demonstrated that miR-210 may be a potential OSAHS biomarker and therapeutic target, and betulin may be a potential candidate for further clinical investigation. Indirect evidence from related disease models suggests that SREBP2-mediated cholesterol accumulation and iron-sulfur cluster disruption contribute to mitochondrial dysfunction in cardiovascular complications [190,191]; however, the specific mechanism by which betulin protects mitochondrial function through SREBP2 inhibition in CIH-induced cardiovascular injury models lacks direct in vivo evidence, representing a promising avenue for future research.

#### 4.4.3 TD139

TD139, a selective Galectin-3 antagonist, has demonstrated direct evidence in mitigating OSAHS-related neuroinflammation, primarily through anti-inflammatory mechanisms; its direct impact on mitochondrial function remains unclear.

TD139 acts as a selective antagonist targeting galectin-3 (Gal-3), exhibiting robust binding affinity toward the protein’s carbohydrate recognition domain. As a β-galactoside-specific lectin, Gal-3 is primarily synthesized in cardiomyocytes and macrophages before being released into the systemic circulation, where it fulfills a pivotal function in inflammatory responses [192].

Direct evidence from CIH models indicates that TD139 markedly represses the elevated expression of NLRP3, while simultaneously lowering the production of pro-inflammatory cytokines IL-1β, IL-8, and the oxidative stress marker MDA, and elevating the levels of the antioxidant enzymes SOD and CAT [31]. However, the endpoints measured in this study are neuroinflammation and oxidative stress rather than mitochondrial function per se. Indirect evidence from related disease models suggests that galectin-3 is associated with cognitive dysfunction [193], and the inhibition of Gal-3 can alleviate diabetes-related cognitive impairment, oxidative stress and neuroinflammation [194].

Observational clinical evidence indicates that Galectin-3 is significantly increased in patients with OSAHS, participating in the development of cognitive impairment [31]. Although Gal-3 is mechanistically linked to apoptotic signaling and neuroinflammatory cascades which intersect with mitochondrial integrity, direct evidence linking TD139 to specific subtypes of mitochondrial dysfunction in OSAHS/CIH models is limited. Further studies are needed to elucidate whether Gal-3 inhibition can ameliorate mitochondrial membrane permeabilization, respiratory chain impairment, or autophagic dysregulation in OSAHS models. Currently, no interventional clinical evidence in OSAHS patients evaluating mitochondrial or neuroinflammatory outcomes is available.

#### 4.4.4 miR-146a-5p

miR-146a-5p, an inflammation-associated microRNA, has shown potential in modulating the HIF1α/mtROS pathway and neuroinflammation in CIH models, representing an emerging epigenetic target; its direct link to specific subtypes of mitochondrial dysfunction requires further validation.

miR-146a-5p is a miRNA derived from microglia exosomes. MiRNAs, a class of small noncoding endogenous RNAs, regulate gene expression by interfering with RNA translation and may be a promising biomarker of candidate molecules [195]. MiR-146a-5p, an inflammation-associated miRNA, is implicated in regulating Alzheimer’s disease alongside the advancement of cognitive deficits and depression in animal models. Prior research has shown that miR-146a-5p expression levels fluctuate following CIH therapy, with elevated levels inducing the generation of the NLRP3 inflammasome and pro-inflammatory mediators—all processes mediated by the HIF1α/mtROS pathway. When miR-146a-5p was increased in microglial vesicles, neuroinflammation was suppressed and cognitive issues improved in rodents subjected to periodic oxygen deprivation [32]. Through its modulation of the HIF1α/mtROS pathway, miR-146a-5p may indirectly influence mitochondrial redox homeostasis and associated inflammatory cascades. Although direct evidence linking miR-146a-5p to specific subtypes of mitochondrial dysfunction (e.g., respiratory chain impairment, permeability transition pore opening, or dynamics dysregulation) remains limited, its regulatory role in neuroinflammatory and oxidative stress responses suggests it may participate in broader mechanisms of mitochondrial stress adaptation. Thus, miR-146a-5p represents a potential therapeutic target and warrants further investigation in the context of CIH-induced mitochondrial and cognitive pathology.

#### 4.4.5 Pseudoginsenoside GQ

Pseudoginsenoside GQ has demonstrated direct but preliminary protective effects in models of OSAHS-related neurological inflammation via anti-inflammatory pathways; its specific impact on mitochondrial parameters warrants deeper investigation.

Pseudoginsenoside GQ (PGQ) is a novel oleanane-type saponin generated via oxidative cyclization of the side chain within 20(S)-ginsenoside Rg3. Ginsenoside Rg3 is extracted chiefly from red ginseng, a traditional medicinal herb with various anticancer, antioxidant, and anti-inflammatory activities [196]. Studies have indicated that PGQ is more stable than Rg3 in plasma and is difficult to hydrolyze during metabolism [197]. PGQ reduces M1 microglial polarization and neurological inflammation induced by CIH through the HMGB1/TLR4/NF-κB pathway [33]. Moreover, PGQ suppresses neuronal apoptosis in the hippocampus and inhibits the production of ROS and inflammatory mediators in the microglia of CIH mice. Therefore, PGQ may be a useful target for treating OSAHS-associated neurological disease. However, it is crucial to interpret these findings with caution, as the current evidence base for its therapeutic potential in the context of OSAHS remains preliminary and limited to a small number of preclinical studies. While these initial reports are promising and provide a mechanistic rationale, they underscore the need for more extensive and independent validation in robust models of OSAHS-related neurological and systemic damage, particularly focusing on its potential effects on mitochondrial bioenergetics, neuroinflammatory cascades, and synaptic integrity.

#### 4.4.6 MCC950

MCC950, a highly selective NLRP3 inflammasome inhibitor, has shown direct efficacy in a model of OSAHS-related renal injury, likely influencing mitochondrial-related stress responses indirectly through inflammation modulation.

MCC950 is a highly selective small-molecule compound that effectively targets and inhibits the NLRP3 inflammasome pathway. Our research revealed that MCC950 binds directly to the Walker B motif in NLRP3’s NACHT domain, thereby blocking ATP hydrolysis and subsequently suppressing inflammasome assembly [198]. Therefore, MCC950 may be a promising new target for the treatment of inflammatory diseases. Moreover, blocking of the NLRP3 inflammasome may play a role in the amelioration of renal injury. In the CIH group, the administration of MCC950 significantly suppresses the upregulation of IL-1β and IL-18 while reducing excessive LDH and ROS production. Studies demonstrated that MCC950 therapy effectively counteracts the elevated expression of Caspase-1, GSDMD, TLR4, MyD88, NF-κB, and NLRP3 in renal tissue, thereby preventing apoptosis in kidney tubular epithelial cells [34]. CIH triggers pyroptosis in the renal tubular epithelial cells by activating the NLRP3 inflammasome. Nonetheless, the use of MCC950, a targeted NLRP3 inhibitor, effectively nullifies the pathological transformations in rats and significantly alleviates renal dysfunction. Through modulation of the NLRP3 pathway, MCC950 may indirectly influence mitochondrial-related inflammatory signaling and cellular stress responses, though its direct effects on mitochondrial function—such as membrane permeability, redox homeostasis, or quality control mechanisms—warrant further investigation in the setting of OSAHS-related organ injury.

#### 4.4.7 anti-miR-155

Inhibition of miR-155 has demonstrated direct evidence in alleviating OSAHS-related renal injury by regulating the NLRP3 pathway; its effects on mitochondria are likely indirect via this inflammatory axis.

MiR-155 is one of the most studied miRNAs that regulates protein expression and has been implicated in diverse cellular processes [199]. MiR-155 inhibitors involve both small molecules and antisense oligonucleotides, which are used to regulate the functions of miRNA-155 [200]. Elevated miR-155 enhances caspase-1, IL-1β, and IL-18 levels, while its inhibitor reduces HK-2 cell inflammation and inhibits renal pyroptosis.

Direct evidence from OSAHS/CIH models indicates that the application of miR-155 inhibitors lessens the activation of the NLRP3 inflammasome within renal tubular cells; conversely, the upregulation of miR-155 intensifies the NLRP3 pathway and exacerbates oxidative stress, largely via the miR-155/FOXO3a/NLRP3 axis [35]. Given the established link between NLRP3 inflammasome activation and mitochondrial dysfunction, miR-155 may indirectly influence mitochondrial homeostasis via this axis, potentially affecting processes such as redox imbalance, inflammatory signaling, and cellular stress responses. Further research is needed to determine whether miR-155 modulation directly impacts mitochondrial bioenergetics, membrane integrity, or quality control pathways in the context of CIH-induced renal injury.

#### 4.4.8 Digoxin

Digoxin, an HIF-1α inhibitor, has provided direct mechanistic evidence in a CIH model of pancreatic injury; however, there is no clear evidence supporting its efficacy in alleviating diabetes, and its direct effects on mitochondria remain undefined.

Digoxin is a hypoxia-inducible factor 1 (HIF-1) inhibitor belonging to a group of cardiac glycosides, which inhibits HIF-1α protein synthesis and expression. Direct evidence from OSAHS/CIH models indicates that digoxin therapy effectively inhibits HIF-1α expression while diminishing ROS production caused by intermittent hypoxia in pancreatic β-cells. The ROS generated through NOX4 activity appears to stimulate excessive insulin secretion, potentially contributing to cellular impairment. Experimental studies in adult mice demonstrated that digoxin administration successfully suppresses HIF-1α levels and prevents the hypoxia-induced elevation of NOX4 at both transcriptional and translational levels, including its enzymatic function [36]. This study utilized digoxin as a specific and critical pharmacological tool to validate the central role of HIF-1α in adipocyte dysfunction and systemic insulin resistance, identifying HIF-1α as a potential therapeutic target for improving insulin sensitivity. It must be clarified that there is currently no clear scientific evidence to support that digoxin can directly or indirectly alleviate type 2 diabetes or insulin resistance.

#### 4.4.9 Melatonin

Melatonin has shown direct protective effects in a CIH model of metabolic dysfunction, potentially through its antioxidant properties and possible modulation of mitochondrial function; however, its dosing and timing require careful consideration due to potential effects on glucose homeostasis.

Melatonin, a neuroendocrine hormone secreted primarily by the pineal gland, plays a key role in regulating circadian rhythms and the sleep-wake cycle. Beyond its chronobiological functions, it also exhibits antioxidant, anti-inflammatory, and immunomodulatory properties [201]. Some studies suggest that melatonin may improve insulin sensitivity and confer metabolic benefits in individuals with diabetes, largely attributed to its antioxidant and anti-inflammatory effects [202]. However, other research indicates that high doses of melatonin or administration at non-physiological times may adversely affect glucose homeostasis [203,204]. Given its role in modulating sleep and circadian rhythms, melatonin has also garnered attention for its potential therapeutic application in OSAHS. A study conducted under CIH conditions revealed that melatonin improves insulin resistance by scavenging ROS/RNS, which in turn increases nitric oxide (NO) bioavailability, leading to vascular relaxation, restoration of microcirculatory perfusion, and enhanced delivery of oxygen and glucose [37].

#### 4.4.10 Sodium-glucose Co-Transporter 2 (SGLT2) inhibitors

SGLT2 inhibitors, with established clinical use in heart failure and diabetes, have shown direct evidence of improving sleep parameters and quality of life in OSAHS patients; their cardiorenal benefits are strongly linked to mitochondrial protection pathways (e.g., AMPK/SIRT1 activation, mitophagy enhancement) in non-OSAHS models, but direct validation of these mitochondrial effects in dedicated CIH models is currently lacking.

SGLT2 inhibitors, initially developed for glycemic control, have emerged as promising agents in managing OSAHS-related comorbidities, particularly cardiovascular disease and metabolic dysfunction, with a compelling mechanistic link to mitochondrial protection.

Clinical evidence from OSAHS patients indicates that SGLT2 inhibitors, such as dapagliflozin, can improve sleep parameters (e.g., Apnea-Hypopnea Index, oxygen desaturation index), quality of life, and reduce systemic inflammation [38,39]. Furthermore, a randomized controlled trial demonstrates that combining dapagliflozin with vitamin D3 provides synergistic benefits for metabolic parameters, nocturnal heart rates, and heart rate variability in hypertensive obese OSAHS patients [40]. Indirect evidence from non-OSAHS models suggests that SGLT2 inhibitors exert cardiorenal benefits by targeting mitochondrial dysfunction and oxidative stress. Proposed pathways include the activation of nutrient deprivation signaling (AMPK/SIRT1), enhancement of mitophagy and mitochondrial biogenesis, reduction of mitochondrial ROS production, and inhibition of the NLRP3 inflammasome—processes central to the pathology of CIH [205,206]. Clinically, SGLT2 inhibitors are well-established for heart failure and chronic kidney disease, with ongoing investigation into their effects in OSAHS-specific populations. However, key limitations include the absence of direct experimental data validating their mitochondrial protective effects in dedicated CIH animal models, a current reliance on theoretical pathway homology rather than direct proof within the OSAHS context, and a need for comparisons with other mitochondrial-targeted therapies (e.g., Mito-TEMPO) to define their unique therapeutic niche.

## 5 Perspectives and conclusions

With the increasing incidence of OSAHS and its associated comorbidities, high economic impacts, such as medical care, emergency treatment, and therapy have increased globally. Current non-pharmacological approaches in the management of OSAHS and its comorbidities—including continuous positive airway pressure (CPAP) therapy, regular physical activity, and weight management—can mitigate disease progression and reduce the risk of complications by ameliorating mitochondrial dysfunction and oxidative stress. However, several limitations persist with these existing non-drug interventions. First, long-term adherence to CPAP is often suboptimal, as many patients discontinue use due to discomfort, device noise, or skin-related adverse effects. Similarly, sustained engagement in exercise and weight control requires considerable self-discipline, adequate baseline health, and consistent allocation of time and resources, making implementation challenging in practice, with outcomes varying significantly among individuals. Second, non-pharmacological strategies primarily influence mitochondrial and oxidative stress states through indirect means, such as improving ventilation, alleviating intermittent hypoxia, or modulating metabolism. In contrast, targeted pharmacological agents may enable more precise intervention at the molecular and cellular levels. By directly regulating mitochondrial function and enhancing antioxidant defenses, such drugs have demonstrated stronger cytoprotective effects in preclinical models—an outcome seldom achievable through non-pharmacological means alone. Furthermore, targeted pharmacotherapy offers the potential for integrated management of multiple comorbidities. Given that mitochondrial dysfunction and oxidative stress represent common pathological mechanisms underlying various OSAHS-related conditions, a single agent acting on these pathways may confer multi-system benefits, thereby simplifying treatment regimens and improving overall therapeutic efficiency, although this hypothesis requires clinical validation. Lastly, early pharmacological intervention targeting these core pathways may impact the natural course of OSAHS-related organ damage, providing not only symptomatic relief but also potential disease-modifying effects, pending confirmation in future studies. Therefore, advancing drug development focused on mitochondrial dysfunction and oxidative stress is crucial for addressing unmet clinical needs and enhancing treatment precision in patients with OSAHS and its associated complications. Accordingly, in the final section of this article, we summarize existing research on relevant targeted therapeutics.

First, we reviewed the roles of mitochondrial dysfunction and oxidative stress in OSAHS-related comorbidities. Although CIH represents the upstream OSAHS-related trigger, the causal hierarchy between mitochondrial injury and oxidative stress after CIH exposure remains incompletely defined because these processes become reciprocal and self-amplifying. It is evident that their interaction contributes to a decline in mitochondrial quality and exacerbates oxidative damage within cells, ultimately leading to cell death. This insight holds significant importance for understanding the mechanisms underlying the development and progression of OSAHS comorbidities. Notably, we observed that most studies on OSAHS have focused on either mitochondrial dysfunction or oxidative stress in isolation, highlighting the need for more research to elucidate the interplay between these two processes. Furthermore, we found that the pathological mechanisms vary across different organ systems in OSAHS-related comorbidities. For instance, in the cardiovascular system, mitochondrial dysfunction appears to play a dominant role, likely due to the heart’s high energy demands, its reliance on a well-organized mitochondrial network, and the precise calcium cycling required for excitation-contraction coupling. Therefore, future efforts could focus on identifying the predominant mechanisms in each affected system and developing targeted therapies based on these mechanisms, thereby advancing precision medicine for OSAHS comorbidities.

Across these organ systems, several shared molecular pathways emerge as central mediators of CIH-induced injury. The NLRP3 inflammasome is consistently activated across cardiovascular, neurological, renal, and pulmonary tissues, serving as a common downstream effector of mtROS-driven inflammation. NF-κB signaling is similarly engaged in multiple systems, driving pro-inflammatory cytokine production and amplifying oxidative stress. The Nrf2/HO-1 antioxidant pathway represents a conserved endogenous defense mechanism, with its activation observed in both pulmonary and cardiovascular models. NO/eNOS signaling is prominently affected in the vasculature and heart, where CIH-induced oxidative stress reduces NO bioavailability and impairs endothelial function. Mitochondrial dynamics regulators, particularly Drp1 and Mfn2, show recurrent dysregulation across cardiovascular, renal, and neurological tissues, indicating that fission/fusion imbalance is a universal feature of CIH-induced mitochondrial injury. Finally, the SIRT1/AMPK signaling axis, which governs mitochondrial bioenergetics and autophagy, is modulated in both cardiac and metabolic tissues. The convergence of these pathways across multiple organ systems underscores the systemic nature of CIH-induced mitochondrial dysfunction and oxidative stress, and supports the rationale for developing therapeutic strategies that target these shared nodes.

Finally, we summarized potential targeted drugs that modulate mitochondrial dysfunction and oxidative damage in OSAHS models, along with their structures and mechanisms of action. Firstly, we observed that most drug research has focused on cardiovascular and neurological disorders in OSAHS patients, whereas studies targeting OSAHS-related diabetes and pulmonary diseases are relatively scarce. Secondly, as many drugs act on a single target, they can only alleviate damage caused by either mitochondrial dysfunction or oxidative stress. However, due to the bidirectional interaction between these two processes, some pathological effects may remain unresolved. Therefore, to improve therapeutic efficacy, it is necessary to develop dual-target agents that concurrently address both mitochondrial dysfunction and oxidative damage, or to evaluate combination therapies targeting both pathways. Moreover, despite the compelling preclinical rationale, a major translational gap remains. It is crucial to note that currently, there are no U.S. Food and Drug Administration (FDA)-approved drugs specifically targeting mitochondrial dysfunction and oxidative stress for the treatment of OSAHS-associated complications. The evidence summarized herein originates almost exclusively from in vitro and animal studies. To date, robust clinical trial data, particularly Phase III trials, supporting the efficacy and safety of these targeted approaches in OSAHS patient populations are lacking. This underscores the imperative for future research to prioritize well-designed early-phase clinical trials to validate these mechanisms in humans, define appropriate patient subgroups, and establish clear efficacy endpoints. Bridging this gap is essential for moving these promising strategies from the laboratory to the clinic.

As a future perspective, it is worth briefly noting that enabling drugs to specifically target relevant systems while minimizing off-target effects represents a key focus for future drug development. Current research indicates that drug targeting may be enhanced through delivery systems, such as nanoparticles, which concentrate therapeutic agents in specific organs. These delivery platforms, including liposomes and polymeric nanoparticles, may achieve passive or active targeting of pathological systems, thereby reducing nonspecific damage to healthy tissues. For OSAHS therapy, ligand-decorated nanoparticles (e.g., conjugated with mitochondrial-penetrating peptides like [SS peptides] or triphenylphosphonium, TPP+) may facilitate the targeted delivery of antioxidants (e.g., CoQ10, Mito-TEMPO analogues) or gene modulators (e.g., PGC-1α mRNA) to metabolically stressed cells in key organs (e.g., cardiomyocytes, neurons, hepatocytes). This approach aims to maximize therapeutic efficacy at the mitochondrial site of injury while minimizing systemic off-target effects. Furthermore, stimulus-responsive systems (e.g., pH- or ROS-sensitive nanocarriers) could be engineered for triggered release specifically within the hypoxic or high-ROS microenvironment of OSAHS-affected tissues. Key Challenges: However, major hurdles remain, including ensuring long-term biosafety and biodegradability of nanomaterials, and achieving efficient penetration across biological barriers (e.g., the blood-brain barrier for neuroprotection), preventing opsonization and rapid clearance, and scaling up GMP-compliant manufacturing. Addressing these challenges through interdisciplinary research is a prerequisite for realizing the clinical potential of these sophisticated delivery platforms in OSAHS management.

Translating promising preclinical findings on mitochondrial and oxidative stress targets into effective clinical treatments for OSAHS requires resolving several critical scientific challenges. Foremost are: (1) Precision Drug Delivery: How can we engineer and verify targeted delivery systems that direct therapies specifically to affected organs or even subcellular compartments like mitochondria, thereby optimizing therapeutic impact at disease sites while reducing whole-body side effects? (2) Human Safety and Durability: What robust methodologies are needed to systematically assess the chronic safety profiles, tissue distribution patterns, and potential adverse reactions of these innovative compounds—particularly mitochondria-specific agents—in human subjects over extended periods?

Closing the persistent disconnect between mechanistic insights from preclinical studies and real-world clinical translation requires targeted prioritization of key strategic research directions, outlined below: (1) Multi-Target Therapeutic Agent Development: Rational design and preclinical validation of next-generation compounds capable of concurrent modulation of interlinked pathological cascades. Single-molecule therapeutics, for instance, that integrate mitochondrial ROS scavenging with upregulation of endogenous antioxidant defense systems—most notably through Nrf2 signaling—or that link augmented mitophagy flux to robust anti-inflammatory activity, may deliver improved therapeutic outcomes by engaging multiple critical checkpoints within the disease progression cascade. (2) Discovery and Clinical Validation of Dynamic Biomarkers: The identification of highly sensitive, specific, and non-invasive biomarkers capable of capturing real-time alterations in mitochondrial function and oxidative stress burden in individuals living with OSAHS. This work encompasses biomarker mining within circulating extracellular vesicles, peripheral plasma, as well as signals captured via state-of-the-art in vivo imaging modalities. These analytical platforms hold indispensable value for enabling early disease detection, accurate patient stratification, and unbiased, quantitative monitoring of treatment response in clinical settings. (3) Personalized and Phenotype-Guided Therapeutic Strategies: Systematic investigation into the crosstalk between an individual’s genetic and epigenetic profile—including functional variants that modulate mitochondrial performance, endogenous antioxidant reserve, and hypoxia response pathways—and distinct OSAHS clinical phenotypes. This line of research will facilitate the stratification of patient subsets that derive maximum clinical benefit from defined mitochondrial-targeted interventions, advancing the broader implementation of precision medicine in OSAHS clinical care.

## CRediT authorship contribution statement

**Linyi Zhong:** Writing – original draft, Supervision, Methodology, Investigation, Conceptualization. **Yiwen Guo:** Supervision, Investigation. **Jiaxuan Zong:** Validation. **Xiushan Yang:** Validation. **Jiaxing Sun:** Validation, Funding acquisition. **Yinwen Zhang:** Validation. **Jiyuan Fan:** Validation. **Yuanhe Feng:** Validation. **Siwen Wang:** Validation. **Yining Wang:** Validation. **Ke Du:** Writing – review & editing. **Mengmeng Wang:** Writing – review & editing, Funding acquisition.

## Funding

This study was supported by grants from the Liaoning Provincial Science and Technology Plan Joint Program (No. 2025JH2/101800382) to Mengmeng Wang and from the Liaoning Provincial Science and Technology Plan Joint Program (No. 2023JH2/101700192) to Jiaxing Sun.

## Declaration of competing interest

The authors declare that they have no known competing financial interests or personal relationships that could have appeared to influence the work reported in this paper.

## Data Availability

No data was used for the research described in the article.
